# Transcriptional analysis of genes involved in competitive nodulation in *Bradyrhizobium diazoefficiens* at the presence of soybean root exudates

**DOI:** 10.1038/s41598-017-11372-0

**Published:** 2017-09-08

**Authors:** Yao Liu, Xin Jiang, Dawei Guan, Wei Zhou, Mingchao Ma, Baisuo Zhao, Fengming Cao, Li Li, Jun Li

**Affiliations:** 1 Institute of Agricultural Resources and Regional Planning, Chinese Academy of Agricultural Sciences, Beijing, 100081 China; 2 Laboratory of Quality&Safety Risk Assessment for Microbial Products (Beijing), Ministry of Agriculture, Beijing, 100081 China

## Abstract

Nodulation competition is a key factor that limits symbiotic nitrogen fixation between rhizobia and their host legumes. Soybean root exudates (SREs) are thought to act as signals that influence *Bradyrhizobium* ability to colonize roots and to survive in the rhizosphere, and thus they act as a key determinant of nodulation competitiveness. In order to find the competitiveness-related genes in *B. diazoefficiens*, the transcriptome of two SREs treated *B. diazoefficiens* with completely different nodulation abilities (*B. diazoefficiens* 4534 and *B. diazoefficiens* 4222) were sequenced and compared. In SREs treated strain 4534 (SREs-4534), 253 unigenes were up-regulated and 204 unigenes were down-regulated. In SREs treated strain 4534 (SREs-4222), the numbers of up- and down-regulated unigenes were 108 and 185, respectively. There were considerable differences between the SREs-4534 and SREs-4222 gene expression profiles. Some differentially expressed genes are associated with a two-component system (i.g., *nodW*, *phyR*-σ^EcfG^), bacterial chemotaxis (i.g., *cheA*, unigene04832), ABC transport proteins (i.g., unigene02212), IAA (indole-3-acetic acid) metabolism (i.g., *nthA*, *nthB*), and metabolic fitness (i.g., *put*.), which may explain the higher nodulation competitiveness of *B. diazoefficiens* in the rhizosphere. Our results provide a comprehensive transcriptomic resource for SREs treated *B. diazoefficiens* and will facilitate further studies on competitiveness-related genes in *B. diazoefficiens*.

## Introduction

Rhizobia could infect legumes roots where they form nitrogen-fixing root nodules and differentiate into intracellular nitrogen-fixing bacteroids. Bacteroids convert molecular nitrogeninto ammonium, which improves host plant growth, and the plant provides dicarboxylic acids, which are photosynthetically fixed carbon molecules, to rhizobia as a source of carbon, energy, and reductants^1^. This microbe-plant interaction is the most efficient and productive source of nitrogen fixation, and has a critical potential for use in sustainable agricultural programs, agriculture, especially if it can be optimized^2, 3^. Due to its higher capacity for N_2_ fixation and increased adaptation to host rhizospheres compared to other rhizobial strains, it has been broadly used in commercial inoculants since 1992^4–7^. However, more than 90% of the rhizobial inoculants introduced into field soils have had minimal effects on nodulation of the developing plants because of their low competitiveness or failure to compete with native rhizobial strains^8^.

For this reason, studies on the mechanism underlying competitiveness may play a key role in the development of commercial inoculants with highly efficient N fixation rates that can be used to improve crop productivity. Rhizobium-legume symbiosis is highly host-specific and is determined by the exchange of diffusible species-specific signals between a host plant and its symbiotic rhizobium^9–12^. More than100,000 low-molecular mass natural products, commonly known as secondary metabolites, are secreted by plants and they change the chemistry and biology of the rhizosphere. These changes have a positive effect on the microbial colonization of the root and microbial activity in the rhizosphere^12–14^. The distinct responsiveness of rhizobia to signal induction confers specificity on the rhizobia, which initiates the symbiotic nodulation of the legume plant and leads to the selection of a compatible plant host-microsymbiont pair^15, 16^. In response to legume-secreted flavonoids, a mechanism involving the NodD family of transcription activators, enables rhizobia to synthesize symbiosis-specific signal molecules called Nod factors (modified lipo-chito-oligosaccharidesor LCOs) by the nod gene operon *nodYABCSUIJ*, and different species of rhizobia generate a diverse range of Nod factors^17^. In host plants, the secreted Nod factors also could induce signal transduction cascades and lead to nodule formation^18, 19^. After Nod factors have been specifically recognized by kinase-like receptors on the host root epidermal cells, they induce root hair deformation, and result in nodule morphogenesis^20^.

In addition to inducing Nod factor production, legume flavonoids (genistein and daidzein) also act as bacterial growth promoters and chemotactic signals^21^, induce type III secretion machinery^22, 23^, lead to the rapid proliferation of rhizobia^15^, and activate rhizobial quorum sensing systems^24^. These phenotypes could affect the nodulation competitiveness of the rhizobia. Interestingly, several non-flavonoid compounds, such as betaines, xanthones, jasmonates, and phenolic compounds, have been shown to act as nodulation gene inducers in rhizobia, including *B. japonicum* and *B. diazoefficiens* ^25–28^. However, some flavonoids act as anti-inducers (antagonists) of *nod* gene transcriptional activation^15^, which demonstrates that the responses to soybean root exudates are complex. It is well known that mixtures of legume-derived extracellular compounds are thought to act as signals that affect rhizobial competitiveness towards legume plants^29^. However, the mechanisms underlying the corresponding recognition and compatibility responses of rhizobia to these root exudates signals are not well understood, and it has not been economically feasible to apply these rhizobia in the field to increase legume yield. The genome sequences of several rhizobia are now readily available^6, 30, 31^. Therefore, it is now possible to use RNA-seq to investigate gene regulation complexity in host-microbe interactions and obtain a better picture of bacterial behavior under agricultural or natural conditions.

Recently, combined transcriptomic studies on the regulation of symbiotic nitrogen-fixation in rhizobial strains were undertaken for the rhizobial model organism *B. diazoefficiens*. The main aims of these studies were (i) to detect transcriptional-level changes and identify genes that were specifically up/down-regulated between free-living conditions and the symbiotic lifestyle within root nodules^32–34^; (ii) to analyze the global gene expression changes and identify the differentially expressed genes in *B. diazoefficiens* in response to a given compound or environmental stresses^35–38^; (iii) to investigate the genes needed for symbiosis and identify their transcriptional regulators by comprehensively comparing the transcriptional profiles of mutants to that of the wild-type^7, 39–41^; and (iv) to analyze the functional mechanism underlying the specific adaptation of *B. diazoefficiens* to different host plant species^42^. However, only a few studies have investigated the gene expression patterns of rhizobial strains with different nodulation efficiencies, the effects of root exudates, and the role of the genes with altered expression in the specific adaptation to a legume host.

The aim of this study was to investigate and compare the impact of soybean root exudates on the transcriptome profiles of two *B. diazoefficiens* strains, which are characterized by contrasting nodulation competitiveness capacities: *B. diazoefficiens* 4534 occupied 90% of the nodules and *B. diazoefficiens* 4222 only 10% when equal amounts of cells were applied to soybean roots^43^. As a result, we have increased our biological knowledge of the mechanisms that underlying the host-specific mechanisms controlling rhizobial competitiveness in response to root exudate induction, and what physiological changes occur during the initiation of rhizosphere colonization. This study has also provided a useful resource for further detailed analysis of specific genes, and for systems biology investigations of other rhizobium-legume symbiosis systems.

## Results and Discussion

### The establishment of standard conditions

Mixtures of flavonoids and other compounds that are exuded by plant roots are thought to act as signals that affect the competitiveness of rhizobia and symbiotic interactions with legumes^29^. It is known that root exudates are primarily responsible for attracting compatible rhizobia and for the expression of a wide range of genes involved in symbiosis^15^. However, there have been few studies on gene expression patterns in response to root exudates. We performed preliminary experiments to establish a standard technique for sampling. The expression levels of *nod* genes (*nodD1* and *nodC*) were chosen as criteria to evaluate the effective induction of SREs.

At the beginning of this study, three variables were compared: (i) nutrient solution concentrations, i.e., soybean plants were grown in different concentrations of nutrient solution; (ii) time intervals between SRE collection, i.e., SREs were collected from 0, 6, 12, and 16-day-old soybean plants; and (iii) optimal rhizobia exposure to SREs for maximal expression of *nod* genes, i.e., the bacterial cells were harvested after exposure to SREs for 0.25-, 1-, 3-, and 6-h. The results were then used to choose the optimal incubation conditions for this study on the effects of SREs on *nod* gene expression and the rhizobium transcriptome. Both *B. diazoefficie*ns strains were grown to the exponential phase and then incubated with 200 mL SREs.

Treating *B. diazoefficiens* with adventitious root cultures led to an increase in *nod* gene expressions, which means they are effective elicitors that are involved in rhizobia nodulation competitiveness and that they are able to trigger rhizobium nodule primordia in legume plants. Therefore, the SREs obtained by this method could be used to induce the expression of bacterial genes involved in the early establishment of microbe-plant interactions. qPCR showed that the *nod* genes of both strains were all up-regulated in response to root exudates, although there were differences in the rate of gene expression up-regulation. The up-regulation of *nod* genes was greater in *B. diazoefficiens* 4534 than in *B. diazoefficiens* 4222. This result enabled us to determine the optimal conditions for *nod* gene expression. These were that the root exudates were collected from soybean grown in one-third strength modified N-free Rigaud-Puppo solution for 7 days, and that the gene induction period was 1-h. This resulted in the greatest up-regulation of *nodD1* and *nodC* genes (Supplementary Figs S1–S3).

### *De novo* transcriptome assembly

cDNA libraries from *B. diazoefficiens* that had been subjected to the different treatments were separately sequenced on the Illumina HiSeqTM 2000 platform and we obtained a total of 69,082,196 raw RNA-seq reads. After filtering out low quality reads, 65,747,612 of clean reads were produced, which represented a majority of the data and the Q20 score was >98%. Each library contained at least 15,244,134 reads, representing a coverage of 67X when compared to the full transcriptome, which is a density regarded as adequate to perform gene expression analysis^44^. The transcript abundance in the sample was quantified on the basis of the number of sequence reads mapped on the genome of *B. diazoefficiens* 4534 and *B. diazoefficiens* 4222 compared to the total number of mapped reads per sample (Table 1). The all unigenes set was used as a reference transcriptome to annotate and analyze the DEGs (differentially expressed ungenes) between the SRE- and control-treated samples.

**Table 1:** Summary of output statistics from four RNA-seq samples treated with SREs and Control, respectively.

Sample	Total Raw Reads	Total Clean Reads	Total mapping Reads	Total Clean Nucleotides (nt)	≥Q20 (%)	Low quality (%)
Control-4534	16,473,244	15,701,208	14,261,018	1,433,774,612	98.64	4.68
SREs-4534	19,796,404	18,756,810	17,300,227	1,697,431,240	98.68	5.25
Control-4222	17,568,414	16,782,508	16,645,319	1,551,750,057	98.71	4.47
SREs-4222	15,244,134	14,507,086	14,086,817	1,339,123,209	98.70	4.83

In order to gain a better understanding of the transcriptome, functional annotation and classification of all the unigenes was performed. In this study, the unigenes with sequence orientations were aligned against public databases, among which total of 8,072 and 8,233 unigenes in stain 4534 and 4222 were mapped in one or more databases, respectively (Table 2).

**Table 2:** Summary of annotation statistics of all unigenes.

Sample	NR	NT	Swiss-Prot	KEGG	COG	GO	ALL
Stain-4534	8029	7680	7109	3695	5679	5255	8072
Stain-4222	7905	7136	6531	3588	5280	5000	8233

**Figure 1:**
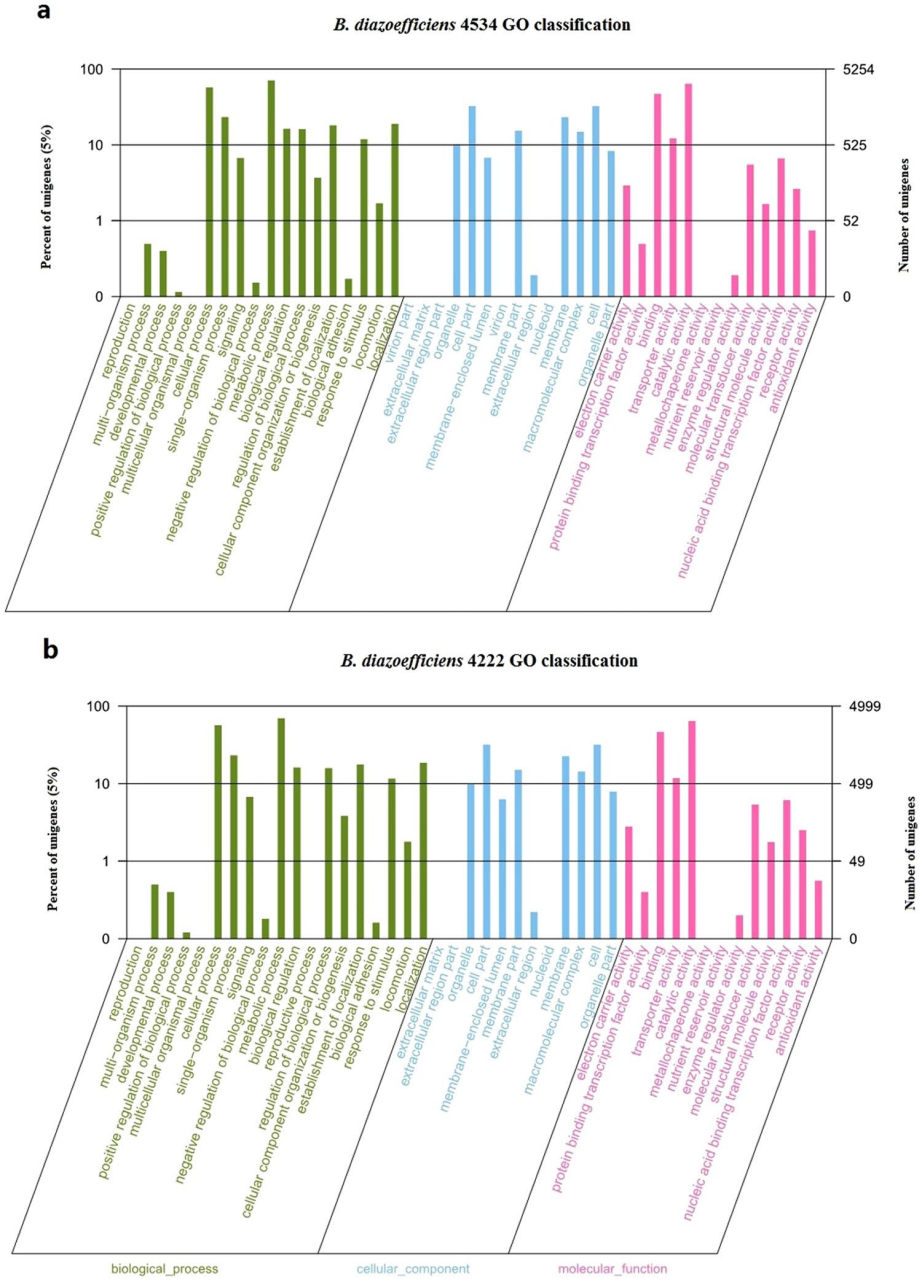
Gene ontology (GO) categories of the unigenes of *B. diazoefficiens*4534 (**a**) and 4222 (**b**). Three kinds of GO categories, biological process, cellular components and molecular functions, are shown on the X-axis. The right Y-axis shows the number of genes in each category, and the left Y-axis shows the percentage.

The functions of all the unigenes were further predicted using GO assignments, a standard system for gene function classification. The unigenes in *B. diazoefficiens* 4534 treated with SREs were mainly assigned to the “cellular process” (3,017) and the “metabolic process” (3,735) subcategories of the “biological process” category, to the “cell” (1,708) and “cell part” (1,708) subcategories of the “cellular component” category, and to the “binding” (2,486) and “catalytic activity” (3,365) subcategories of the “molecular function” category (Fig. 1a). The unigenes in *B. diazoefficiens* 4222 treated with SREs were also assigned to the “cellular process” (2,834), “metabolic process” (3,491), “cell” (1,592), “cell part” (1,592), “binding” (2,322), and “catalytic activity” (3,206) subcategories (Fig. 1b).

Pathway assignment for all transcripts was based on the KEGG database. In response to SREs, a total of 8,072 unigenes in *B. diazoefficiens* 4534 were assigned to 158 KEGG pathways. The analysis indicated that “Metabolic pathways” (486), “Microbial metabolism in diverse environments” (262), “Biosynthesis of secondary metabolites” (243), “ABC transporters” (203), and “Two-component system” (95) were the dominant pathways. Similarly, for SREs treated *B. diazoefficiens* 4222, a total of 8,233 unigenes were annotated and were assigned to 160 KEGG pathways. The dominant pathways were “Metabolic pathways” (390), “Microbial metabolism in diverse environments” (225), “Biosynthesis of secondary metabolites” (207), “ABC transporters” (171) and “Two-component system” (77).

The analysis of the GO and KEGG categories for the unigenes under SREs induction showed similar enrichment terms in each category for SREs-4534 and SREs-4222, which meant that the symbiosis mechanism was very conservative. However, under the same conditions, the number of unigenes in these categories was greater in SREs-4534 than in SREs-4222.We suggest that the differences in *B. diazoefficiens* responses to SREs may result in the differences in symbiotic matching ability. Analyzing the DEGs expressed in *B. diazoefficiens* represents an efficient and simple way to further identify and explore the genes related to nodulation competitiveness.

### Differential transcriptomes of two strains treated with soybean root exudates

**Figure 2:**
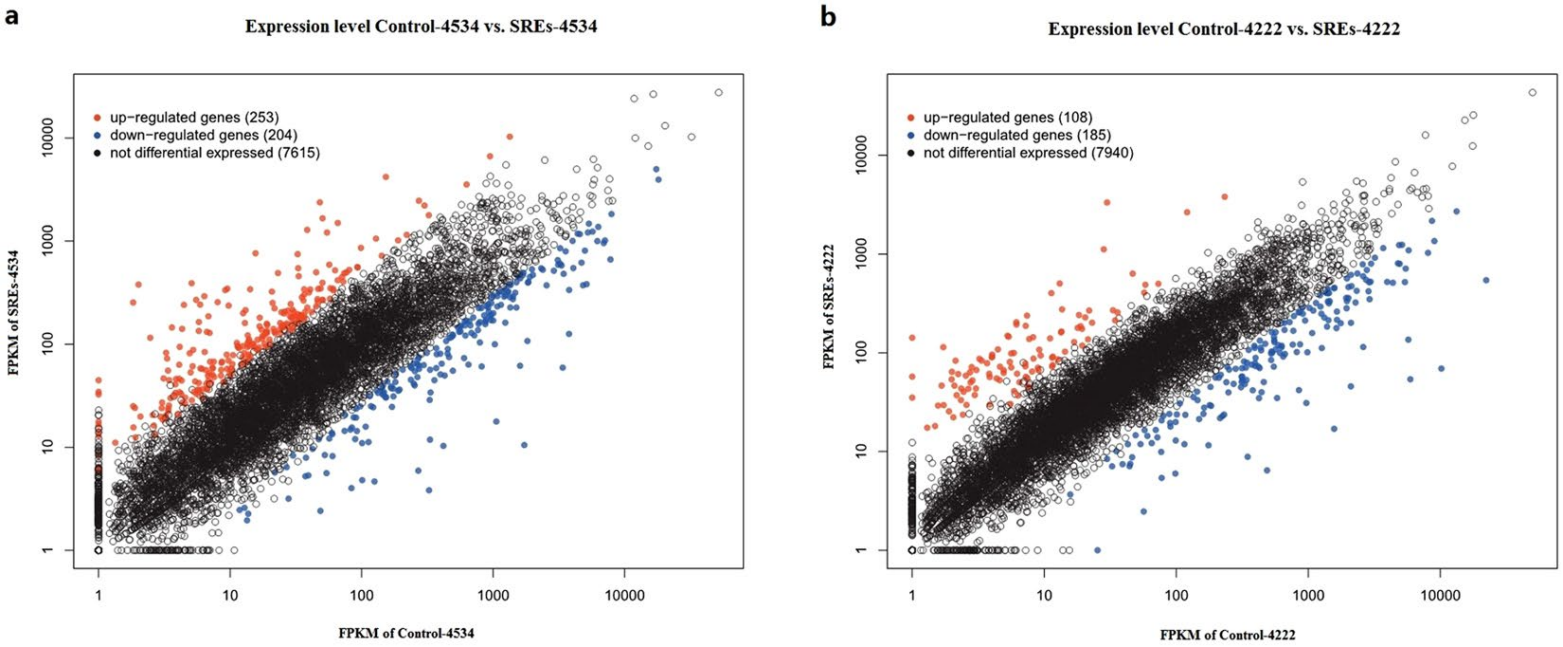
Distribution of differentially expressed genes in *B. diazoefficiens* 4534 (**a**)and 4222 (**b**), respectively.

RNA-seq and a comparative analysis of *B. diazoefficiens* induced by SREs were performed to explore nodulation competitiveness-related genes. The DEGs was assessed between the SREs and control treatments were obtained from a transcriptome data comparison of the Control-4534 vs. SREs-4534 in *B. diazoefficiens* 4534 and Control-4222 vs. SREs-4222 in *B. diazoefficiens* 4222, many genes exhibiting significantly differential expressions were identified. A total of 457 DEGs were identified in SREs-4534(Supplementary Table S1). These consisted of 253 up-regulated and 204 down-regulated genes (Fig. 2a). In SREs-4222, 293 DEGs were identified consisting of 108 up-regulated and 185 down-regulated genes (Fig. 2b) (Supplementary Table S2).

Many assembled unigenes were not significantly matched with available databases due to their short sequences or because they represented significantly novel genes. Analysis of the transcriptome profiles showed that the expression of a number of genes was significantly altered (*P < *0.05) in both stains in response to SREs, which confirmed the effectiveness of the SRE s collection method. To our knowledge, this is the first report on transcriptome data that shows the response of *B. diazoefficiens* to SREs treatment. Therefore, this study will help reveal the molecular mechanisms underlying the different nodulation abilities in *B. diazoefficiens*, and specific adaptations by the legume host. The DEGs were identified under SREs treatment accounts for just a fraction of the total full-length sequences that had been annotated in *B. diazoefficiens*. One of the reasons is that SREs concentrations are very low and only the genes that are responsive to low concentrations of bioactive compounds have been induced^20^. Furthermore, symbiotic interaction in the rhizosphere involves a far more complex series of interactions, such as attachment to roots hairs, growth rate on rhizosphere substrates, contact with complicated plant macromolecules, biofilm formation, and cell-cell competition^20, 45^.

### GO and KEGG enrichment analysis of DEGs

We performed GO and KEGG enrichment analyses to assign functional categories to the DEGs in the two strains treated with soybean root exudates. The DEGs in SREs-4534 were enriched in 12 GO terms while the DEGs in SREs-4222 were enriched in 7 GO terms (Supplementary Fig. S4a,b). The enriched 12 GO terms were “ciliary or flagellar motility”(20), “cell motility”(20), “cellular component movement” (20), “locomotion”(36), “chemotaxis” (18), “taxis”(18), “response to external stimulus”(19), “response to chemical stimulus” (20), “oxygen transport” (11), “gas transport” (11), “cell projection organization”(6), and “flagellum organization” (6) in the “biological processes” category; and “motor activity”(13), “oxygen binding”(11),“structural molecule activity”(17),“oxidoreductase activity”, and “acting on other nitrogenous”(3) in the “processes” category. The seven enriched *B*. *diazoefficiens* 4222 GO terms were “locomotion” (23), “ciliary or flagellar motility” (23), “cell motility” (23), “cellular component movement”(23), “cell projection organization”(8), “flagellum organization”(8), and “bacterial-type flagellum part” (18) in the “biological processes” category. These results suggest that the GO terms associated with “flagellum motility”, “cell motility”, and “flagellum organization and locomotion” were highly enriched in both of stains treated with SREs. Interestingly, GO terms related to “chemotaxis” and “response to external/chemical stimulus” were highly enriched in SREs-4534, but did not significantly change in SREs-4222. This suggested that a series of activities involved in chemotaxis and stimulus response are triggered in B. diazoefficiens 4534 following SREs treatment, and that these could play a role in nodulation efficiency^35^. This is in agreement with a previous report about the proteomic analysis of *B. japonicum*in response to genistein^46^.

In the KEGG pathway enrichment analysis, the genes that encoded enzymes involved in “flagellar assembly” (29), “bacterial chemotaxis” (37), and the “two-component system” (25) were highly enriched in SREs-4534 (Supplementary Fig. S5a). However, pathways corresponding to “flagellar assembly” (30) and “nitrogen metabolism” (11) were found to be highly enriched in SREs-4222 (Supplementary Fig. S5b). Based on analyses of the pathways, most gene expressions, especially genes related to “bacterial chemotaxis” and the “two-component system”, could have roles in the competitiveness of specific *B. diazoefficiens* strains. This is consistent with the results for the GO enrichment analysis and the proteomic analysis of *B. japonicum*in response to genistein.

### Differential gene expression patterns between the two strains treated with SREs

#### Genes associated with the “two-component system”

Many studies have shown that the “two-component system” regulates the specific gene expression of rhizobia by recognizing additional plant and environmental signals, which affects rhizobial ability to colonize roots. It also affects the competitive performance of rhizobia^19, 47, 48^. This study showed that a total of 19 genes were related to the “two-component system”, which was found to be significantly enriched in the KEGG analysis (Supplementary Table S3). They considerably changed their expression in SREs-4534 where 18 genes were up-regulated and one was down-regulated. Interestingly, three genes were involved in associated with the “two-component” regulatory family, and the genes encoding NodW, PhyR and σ^EcfG^ were up-regulated in strain 4534 after SREs treatment, but they did not significantly change in SREs-4222.

Nod W is a two-component response regulator that is phosphorylated at the Asp70 residue by its cognate sensor protein Nod V, which controls the expression of the nodulation genes^35^. In *B. japonicum*, Nod VW is thought to recognize different plant flavonoids and environmental signals, or, when combined with Nod D, it may plays a key role in the nodulation of a broader range of specific host plants by mediating Nod factor synthesis^19^. These results are comparable to our GO enrichment analysis, where the GO term related to the “external/chemical stimulus” response was only highly enriched in SREs treated strain 4534. Furthermore, the Nod VW “two-component system” provides an alternative pathway for nod gene activation In *NodD1* mutants (Faruque) and similar systems have been found in many rhizobia, including, *Sinorhizobium meliloti* (ExoS/ChvI), *Mesorhizobiumloti* (VirA⁄VirG) and *Agrobacterium tumefaciens* (ChvG/ChvI)^49^. Another, PhyR is a “two-component response” regulator that bridges two component signal transduction and is an alternative σ factor regulatory paradigm. It contains an N-terminal σ-like domain and a C-terminal receiver domain^50^. Previous studies have demonstrated that these PhyR and σ^EcfG^ regulons regulators act in the same cascade. Upon sensing a particular stimulus, the PhyR activity is controlled by phosphorylation of a conserved Asp residue and binds NepR, releasing σ^EcfG^ that can then associate with the RNA polymerase core enzyme to transcribe stress-related genes^51^. Subsequently, PhyR/σ^EcfG^ has been found to be involved in the responses to a number of stress and cellular growth signals, including nutrient deficiency, low pH, and heat shock, as well as oxidative, UV, ethanol, and hyperosmotic stresses^52^. PhyR/σ^EcfG^-lacking *B. japonicum* mutants had impaired nodulation abilities. Further research needs to uncover the signal(s) and transduction mechanisms involved in the control of novel symbiotic genes^46^.

When *B. diazoefficiens* was induced by the SREs, a series of “two-component response” regulator (NodW, PhyR and σ^EcfG^) encoding genes were considerably up-regulated, which is consistent with what was previously reported for the comparative proteomic study of *B*. *diazoefficiens* 4534 and 4222 by incubation with SREs. Thus, these regulators had positive effects on the higher nodulation competitiveness of *B*. *diazoefficiens* 4534^53^.

#### Genes associated with bacterial chemotaxis

“Flagella and motility related functional” genes were enriched categories shared by both strains. The DEGs associated with chemotaxis were significantly enriched in SREs-4534 compared to SREs-4222. A total of 17 DEGs were observed in this pathway, all of which were up-regulated and were related to flagella and tropism (Supplementary Table S4). For example, *cheA* was only expressed in SREs-4534, and Donati *et al*. validated the effect of stimulating factors on the tropism and nodulation of rhizobia by knocking out *cheA*, which is consistent with the proteomics study^37^. This indicated that the expression of genes associated with tropism plays important roles in environmental adaptation and selective nodulation by rhizobia^54^.

It has been shown that genes related to flagella and bacterial tropism play essential roles at the beginning of the selective nodulation process in rhizobia^54, 55^. Different rhizobia exhibit different chemotactic responses to the root exudates produced by their hosts^55, 56^. Environmental factors can induce the expression of taxis factors in *Rhizobium leguminosarum*. These taxis gene clusters may affect comparative nodulation capabilities by regulating motility swimming and chemotaxis in *R. leguminosarum* ^57^. Furthermore, proteomic experiments indicated that more abundant flagellar proteins were detected in the strain 4534 than strain 4222 when both were exposed to SREs^53^. And similar result was recorded by Lang when rhizobia responded to genistein^35^. Tropism related genes (e.g., unigene04832) in *B. diazoefficiens* 4534 showed a stronger activity during the response to SREs than strain 4222. We suggest that the differences in the signal transduction and tropism related genes between the strains during the response to root exudates led to the variation in the selective nodulation capabilities of *B. diazoefficiens*. This is probably caused by the direct regulation of some nodulation signals or by an indirect impact on flagella synthesis.

#### Genes associated with ABC-transport proteins

The diverse functions of ABC transport proteins in bacterial physiological processes is due to their adaptability to the environment^58^, but their main active stage is in the early stages of rhizobium symbiotic system establishment^46^. After SREs induction, a significant enrichment of the ABC transport protein associated with DEGs was observed. A total of 20 DEGs were identified in this pathway, all of which were up-regulated (Supplementary Table S5). They were mainly involved in amino acid and carbohydrate transportation. The conclusions are found to be in line with what was previously reported for the comparative proteomic study of *B. diazoefficiens* 4534 and 4222 induced by SREs^53^. For example, biotin metabolism and the folic acid synthesis process were differentially expressed pathways in SREs-4534 according KEGG, and numerous complex intermediate metabolites and other precursors are produced by these processes. Therefore, to ensure the transport of these molecules, a considerable number of transport proteins are encoded by rhizobium species, and the ABC transport protein is the most important one^46^. The study of *B. japonicum* type bacteria demonstrated that only certain ABC transport protein types were expressed during the early stage of rhizobium symbiosis^59, 60^. Transport protein related to amino acid metabolism was one of the important classes, which is consistent with the data for *S. meliloti* type bacteria^61^. Based on the mutation analysis of the specific amino acid ABC transport proteins in *R. leguminosarum*, Lodwig *et al*. proposed a complex mechanism of amino acid recycling for the symbiotic nitrogen fixation process^62^.

These data indicated that the carbohydrate ABC transport system in rhizobium promoted the absorption of nutrients that were only presented at low concentrations in the rhizosphere and soil environment, and that this difference in nutrient uptake possess a strong relation to competitiveness for nodulation^1^.

#### Genes associated with phytohormone metabolism

All known phytohormones affect the nodulation of rhizobium. Auxin and cytokinin act as positive regulators in the formation and development of nodule and ethylene acts as an inhibitor that prevents root infection by rhizobia and nodule formation^6, 63, 64^. Indole-3-acetic acid (IAA) is a plant hormone that can regulate the formation of root nodules, and it plays important roles in many cellular processes, such as nodule formation, stimulation of early cell division, and nodule differentiation^65^. It has been shown that the IAA produced by *Bradyrhizobium* may affect symbiosis with the host^66, 67^, but how this occurs is still unclear. Therefore, IAA produced by rhizobium during the symbiosis process might affect the IAA balance inthe host plant, which would influence the establishment of the rhizobia and host symbiont^6, 65^.

These results demonstrate that two genes related to IAA synthesis, *nthA* encoding nitrile hydratase subunit alpha protein and *nthB* encoding nitrile hydratase subunit beta protein, which constitute nitrile hydratase (Nhase), were up-regulated in SREs-4534. Nitrile hydratase is an enzyme that converts indol-3-acetonitrile into indole-3-acetamide (IAM). Most rhizobia can synthesize IAA via different pathways, and at least five of them have been shown to initiate the synthesis of tryptophan^6, 68, 69^. In this group of synthetic pathways, most is known about the IAM route, where the tryptophan is first converted into IAM in the presence of tryptophan monooxygenase and then converted to IAA by IAM hydrolase^66^. Recent studies have shown that in *Bradyrhizobium*, the synthesis of IAM is independent of TMO activity and related to nitrile hydratase activity^6, 65, 66^. Although the regulation mechanisms for the biosynthesis of IAA from tryptophan in *B. diazoefficiens* have not been fully characterized, it may play a key role in its competitive colonization of rhizospheres. It also suggests that the regulation of IAA in nodules is very complex.

#### Genes associated with metabolic fitness

It is well known that the quantity of single carbon sources in soil is limited and that there are various types of carbon sources. Some special carbohydrates around the root, such as stachydrine, myo-inositol, and homoserine, can be used by rhizobia for growth^70, 71^, which is necessary if symbiotic nodulation and nitrogen fixation are to occur. To a certain extent, the nutrient utilization ability of rhizobium in the soil when faced by limited nutrient resources determines its selectively competitive nodulation capability^72, 73^.

Stachydrine and homoserine are major components of SREs, and they can lead to the production of large amounts of proline after degradation. In this experiment, *putA* expression in strain 4534 was significantly higher than in strain 4222. The proline dehydrogenase encoded by the *putA* gene in *S. meliloti* oxidizes proline into glutamate. Glutamate is then deaminated to produce α-ketoglutarate, which can participate in the citric acid cycle to produce energy or carbohydrate. Therefore, proline is an essential source of energy at the early stage of rhizobium infection. Mutant analysis showed that *putA* played a role in the selective nodulation capability and the colonization ability of rhizobia around host roots^74, 75^. Besides, the results of the proteome analysis showed that under the induction of SREs, more proteins related to metabolic fitness were found up-regulated in *B. diazoefficiens* 4534 than that of in *B. diazoefficiens* 4222^53^.

In addition, the gene encoding prolidase (e.g., unigene05621) was up-regulated in both strains, and it effectively decomposes compounds containing proline residues, which increases the amount of proline that can be utilized by rhizobia. Therefore, we believe that the genes related to metabolism fitness are surely playing an important role in nodulation competition. Rhizobia lability to effectively utilize nutrients in soils containing limited nutrient resources determines the selectively competitive nodulation capabilities between different strains and the host.

### Quantitative real-time PCR (qPCR) verification

**Figure 3:**
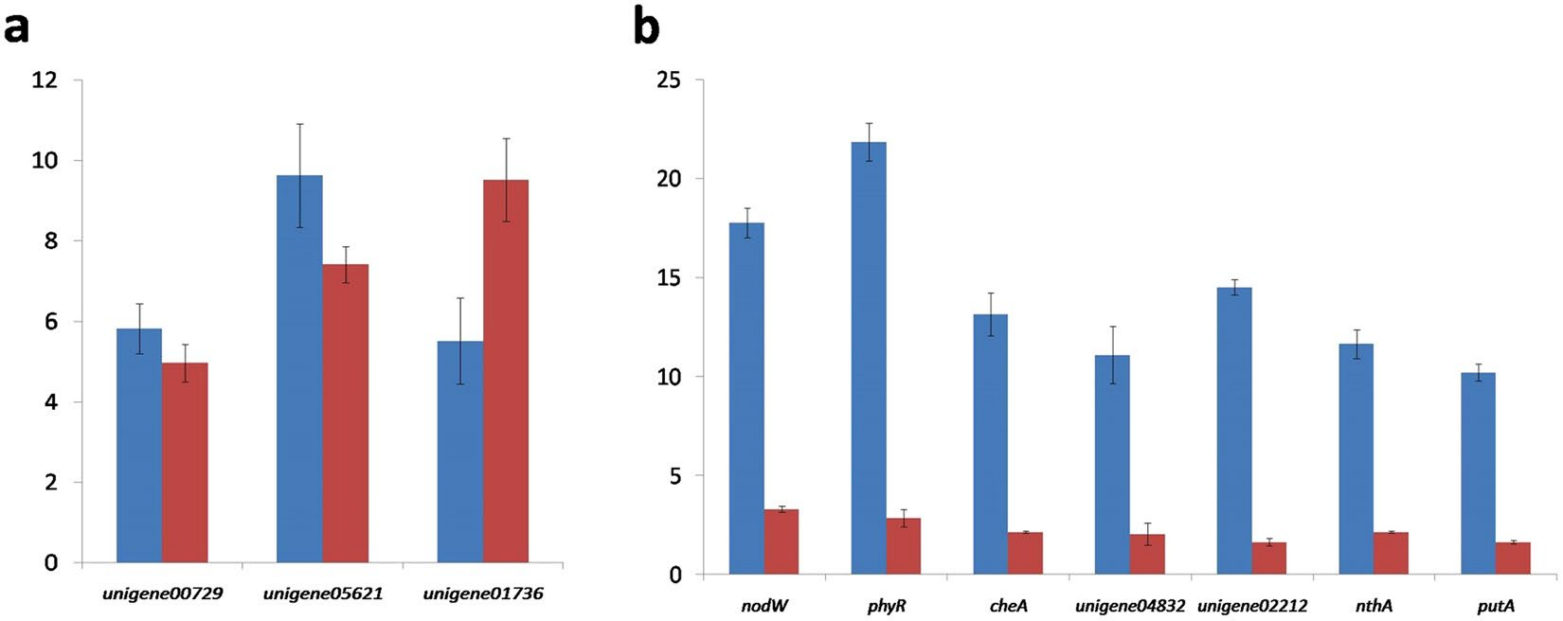
Expression pattern validation of selected unigenes of *B. diazoefficiens* after SREs treatment through qRT-PCR. (**a**) The gene expression was up-regulated in both strains after SREs treatement. (**b**) The gene expression was up-regulated in strain 4534 after SREs treatment, but no significantly change in strain 4222 treated with SREs.Three technical replicates were performed for each of the three biological replicates. The height of each bar chart represents the mean average of sample-specific 2^−ΔΔCt^ values. The bars in blue represent the *B. diazoefficiens* 4534 after SREs treatment, and the bars in red represent the *B. diazoefficiens* 4222 after SREs treatment.

To confirm the accuracy and reproducibility of the results of transcriptome analysis, quantitative PCR analysis was performed on 10 selected DEGs identified from the transcriptome profiling experiments, which divided into two groups according to their expression levels. In the first group, containing three genes encoding a sugar transporter (unigene00729), prolidase (unigene05621) and flagellin (unigene01736), the gene expression was up-regulated in both strains after SREs treatement (Fig. 3a). In another group, the DEGs were up-regulated in SREs-4534, but no significantly change in SREs-4222. The genes encoding two-component system (*nodW*, *phyR*), bacterial chemotaxis (*cheA*, unigene04832), ABC transport proteins (unigene02212), IAA (indole-3-acetic acid) metabolism (*nthA*) and metabolic fitness (*putA*) fitted this pattern (Fig. 3b). The qPCR results of 10 selected genes were consistent with their transcript abundance changes as determined by RNA-seq, which indicated the reliability of the transcriptome data.

## Conclusion

This study is a comparative survey of the impact of SREs on the transcriptome profiles of two *B. diazoefficiens* strains that have different nodulation abilities. It reveals several previously uncharacterized genes involved in the competitive nodulation process. A majority of the genes led to the differential expression of a diverse array of proteins/enzymes, which suggested that there was a considerable variation in metabolic and physiological activities between the two SREs treated strains. This could affect *B. diazoefficiens* competitiveness. The genes involved in a variety of metabolic pathways, such as the two-component system, bacterial chemotaxis, ABC transport proteins, IAA metabolism, and metabolic fitness, were highly expressed in SREs-4534 compared to SREs-4222. When exposure to SREs, we observed that the most DEGs in both *B. diazoefficiens* strain 4534 and 4222 were highly correlated to differentially expressed proteins in the proteomic study^53^. Differential responses to induction by particular compounds in the root exudates may mediate the selection of specific rhizosphere populations. We found that competitive strains more readily responded to a wide range of synthetic flavonoids and seed exudates in comparison to uncompetitive strains.

Some other important DEGs were also validated in previous comparative proteomics analysis of *B. diazoefficiens* treatment with different flavonoids (genistein, coumestrol and daidzein, etc.) as well as the proteome profiles of *B. diazoefficiens* bacteroid during soybean nodule development^46, 76–80^. The data further revealed that the regulation of signaling transduction, receptor-mediated recognition, and the ability to metabolize various nutrient substances and phytohormones at the transcriptional level were fundamental to successful host colonization by rhizobia. Overall, this study has produced new information about the genetic and functional responses of *B. diazoefficiens* during competitive nodulation, and has provided a molecular basis for further investigations into the mechanisms underlying the host-specific symbiosis of bacteria with soybean and other legume hosts.

## Methods

### Bacterial strains, medium, and culture conditions

*B. diazoefficiens* 4534 and *B. diazoefficiens* 4222 were originally isolated from the nodules of field grown soybeans in Henan Province and Anhui Province, China, respectively. The soybean cultivar was Zhong-huang 13, which is widely cultivated in the Huang-Huai-Hai region on the North China Plain. All the rhizobia were pre-cultured at 180 rpm and 30 °C to an optical absorbance at 600 nm in TY medium (5 g/L tryptone, 3 g/L yeast extract, and 1.3 g/L CaCl_2_.6 H_2_O, [pH 7.0])^81^. The cultured cells were harvested, immediately flash frozen in liquid nitrogen, and stored at −80 °C until needed for RNA isolation.

### Seedling growth and preparation of soybean root exudates (SREs)

Mixtures of flavonoids and other compounds that are exuded by plant roots can be more effective at improving the competitiveness of rhizobia than single compound^26^. Soybean seeds were surface-sterilized and germinated on a nitrogen-free mediumin the dark at 28 °C for 24–48 h^5^. Twenty-five pre-germination seeds were transferred to a polypropylene lattice placed in a glass cylinder containing 300 mL of sterilized, modified N-free solution under aseptic conditions^82^. No seeds were sown in the control treatment. The plant and control microcosms were arranged in a replicated randomized block design and maintained at 30 °C for 16 h and then at 15 °C for 8 h. The root exudates and control eluent (liquid from the blank control) were applied on TY solid medium and no growth indicated that no microbial contamination was present. Each collection had three biological replicates.

### Sample preparation for RNA-seq RNA extraction

Total RNA was extracted using the SV Total RNA Isolation System (Promega, Madison, WI, USA) according to the manufacturer’s protocol, and then the genomic DNA was purified with RNase-free DNase I (Promega). The total RNA concentration was assessed using a NanoDrop-100 (Thermo Scientific, Wilmington, USA) and its quality was tested using an Agilent Bioanalyzer 2100 (Agilent Technologies, Santa Clara, CA, USA). All samples had A_260_/A_280_ and A_260_/A_230_ ratios of 2.13–2.23, RIN (RNA Integrity Number) value above 8.5, and 23 S/16 S values above 1.6 except for one.

### Quantitative real-time PCR (qPCR) certification

qPCR as used to detect the effects of root exudate on *nodC*, *nodD1*, and *nodD2* gene expressions and verify the reliability of the transcriptome experiments. Total RNAs were isolated as described previously. The first strand cDNAs were synthesized using1.0 μg of RNA and a ProtoScript First-Strand cDNA Synthesis Kit (New England Biolabs, Ipswich, MA, USA). The RT samples were used for quantitative qPCR with the primers shown in Table 1, which were designed using the Premier 5.0 based on the genomic *B. diazoefficiens* USDA110^83^, which targets an amplicon size of 150–200 bp. The specificity of the primers was certified by agarose gel electrophoresis and product dissociation curves^84^. The expression of 16 S rRNA was used as an internal control for normalization. The equipment used included a 7500 Sequence Detection system (Applied Biosystems, Foster City, CA, USA) and SYBR Green PCR Master Mix (Applied Biosystems).

### RNA-Seq experimental design, library construction, and sequencing

An equal amount (at least 5 μg) of total RNA from each sample was pooled for RNA-Seq so that a comprehensive range of transcripts could be obtained. Isolated mRNAs were extracted using Oligo (dT) magnetic beads and broken down to about 200 bp lengths using fragmentation buffer. The fragmented mRNA was reverse-transcribed into cDNA using a Truseq RNA-seq kit (Illumina Inc., CA, USA) and random primers. After end reparation and the addition of a single nucleotide A (adenine) at the 3′-end, the cDNAs were connected with adapters and prepared as libraries. After qualification and quantification using an Agilent 2100 Bioanaylzer and a StepOnePlus Real-Time PCR System (ABI, CA, USA), their sequences were determined using an Illumina HiSeq™ 2000 (Illumina Inc).

### Transcript assembly and functional gene annotation

The raw data outputs from the Illumina equipment were filtered to remove adaptor sequences, unknown bases higher than 10%, and low quality bases (bases with a quality value ≤ 5), which left just high quality (clean) reads. The clean reads were assembled using the Trinity software short reads assembly program (http://trinityrnaseq.sourceforge.net/). The high quality reads were assembled into contigs, and then integrated to obtain unigenes. Subsequently, the “all unigenes” was assembled from the unigenes of Control-4534, SREs-4534, Control-4222, and SREs-4222, and used as a reference transcriptome for further annotation and analyses.

To obtain annotation information for the transcriptome in *B. diazoefficiens*, the sequences of the unigenes were aligned with the following databases: NR (non-redundant database), NT (nucleotide database), Swiss-Prot (Swiss Protein database), KEGG (Kyoto Encyclopedia of Genes and Genomes), COG (Clusters of Orthologous Groups) and GO (Gene Ontology).

### Analysis of differentially expressed genes (DEGs)

The expression levels of the *B. diazoefficie*ns DEGs in the different treatments were calculated by the RPKM (reads per kilobase per million mapped reads) method. An FDR (false discovery rate) ≤0.001 and a log2 (RPKM-SREs/FPKM-Control) ≥1 were adopted to judge the DEGs between the SREs and the control treatments. To understand the distribution of the DEGs, GO annotations for the DEGs were assigned to three categories (“molecular function”, “biological process”, and “cellular component”) using the Blast2GO software (http://www.geneontology.org). The GO enrichment analysis of the DEGs was undertaken using Goatools software (https://github.com/tanghaibao/GOatools) to find significantly enriched GO terms among the DEGs. Differentially expressed gene enrichment in the KEGG pathways was carried out by KOBAS software (http://kobas.cbi.pku.edu.cn/home.do). The DEGs that were significantly enriched in GO terms and KEGG pathways were identified after hypergeometric testing (Bonferroni-correction *P* ≤ 0.05)^85^.

## Electronic supplementary material

Supplementary Information
