# Supplementary material for: Transcriptional analysis of genes involved in competitive nodulation in Bradyrhizobium diazoefficiens at the presence of soybean root exudates
Source: Sci Rep. 2017 Sep 8;7:10946. doi: 10.1038/s41598-017-11372-0 (PMC5591287; doi:10.1038/s41598-017-11372-0)
Supplement: Supplementary file 1 — Supplementary Information [file 41598_2017_11372_MOESM1_ESM.doc]

**Transcriptional analysis of genes involved in competitive nodulation in *Bradyrhizobium diazoefficiens* at the presence of soybean root exudates**

Yao Liu^1^, Xin Jiang^1,2*^, Dawei Guan^1^, Wei Zhou^1^, Mingchao Ma^1,2^, Baisuo Zhao^2^, Fengming Cao^1,2^, Li Li^1^, Jun Li^1,2*^

*1Institute of Agricultural Resources and Regional Planning, Chinese Academy of Agricultural Sciences, Beijing 100081, China,*

*2Laboratory of Quality&Safety Risk Assessment for Microbial Products (Beijing), Ministry of Agriculture, Beijing 100081, China,*

***Corresponding author: Institute of Agricultural Resources and Regional Planning, Chinese Academy of Agricultural Sciences, Beijing 100081, PR China. Tel: +8610 82106208. FAX: +86 1082108702

E-mail addresses: lijun01@caas.cn.

**Figure S1. Different root exudates acquired from soybean plants grown under various nutrient concentrations and their effect on the expression levels of *nodC* and *nodD1* genes of two different *B. diazoefficiens* strains (4534 and 4222).**


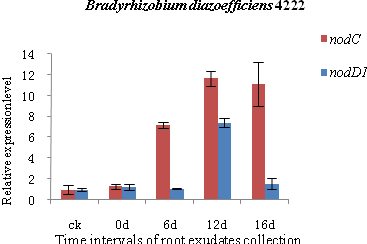

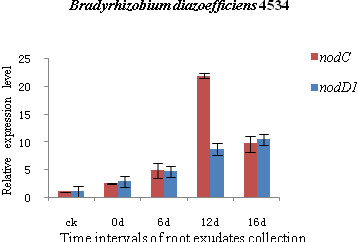


**Figure S2. Root exudates taken at different intervals from soybean plants (0 days being time of first inoculation) growing in optimal nutrient concentration and their effect on the expression levels of *nodC* and *nodD1*** **genes of two different *B. diazoefficiens* strains (4534 and 4222)**


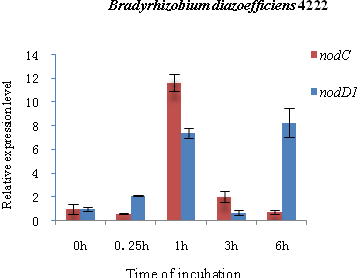

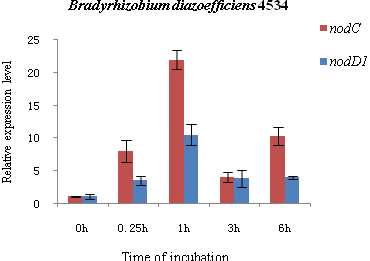


**Figure S3. The effect of different root exudates incubation periods on the expression levels of *nodC* and *nodD1* genes in two different *B. diazoefficiens* strains (4534 and4222)**


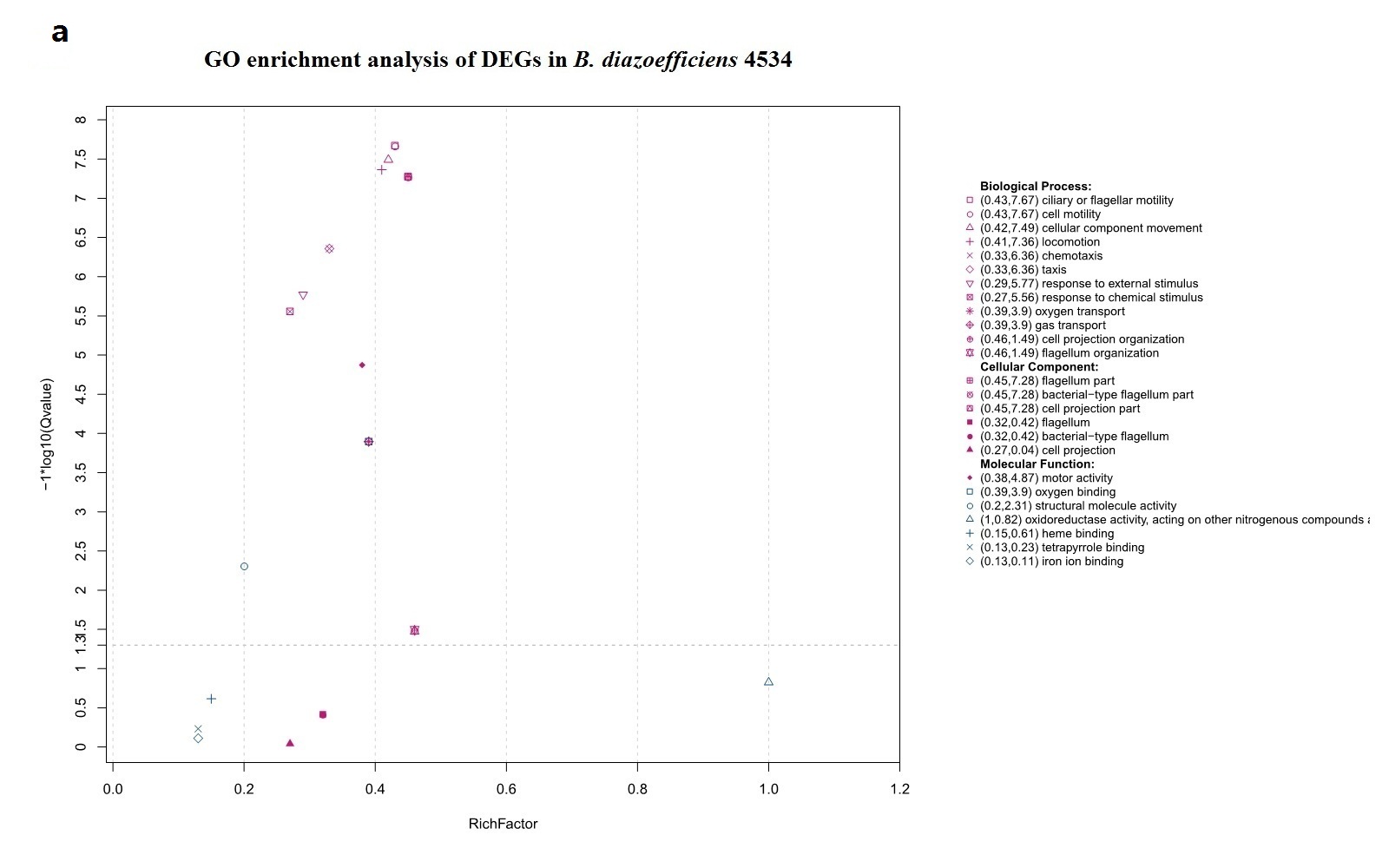


**
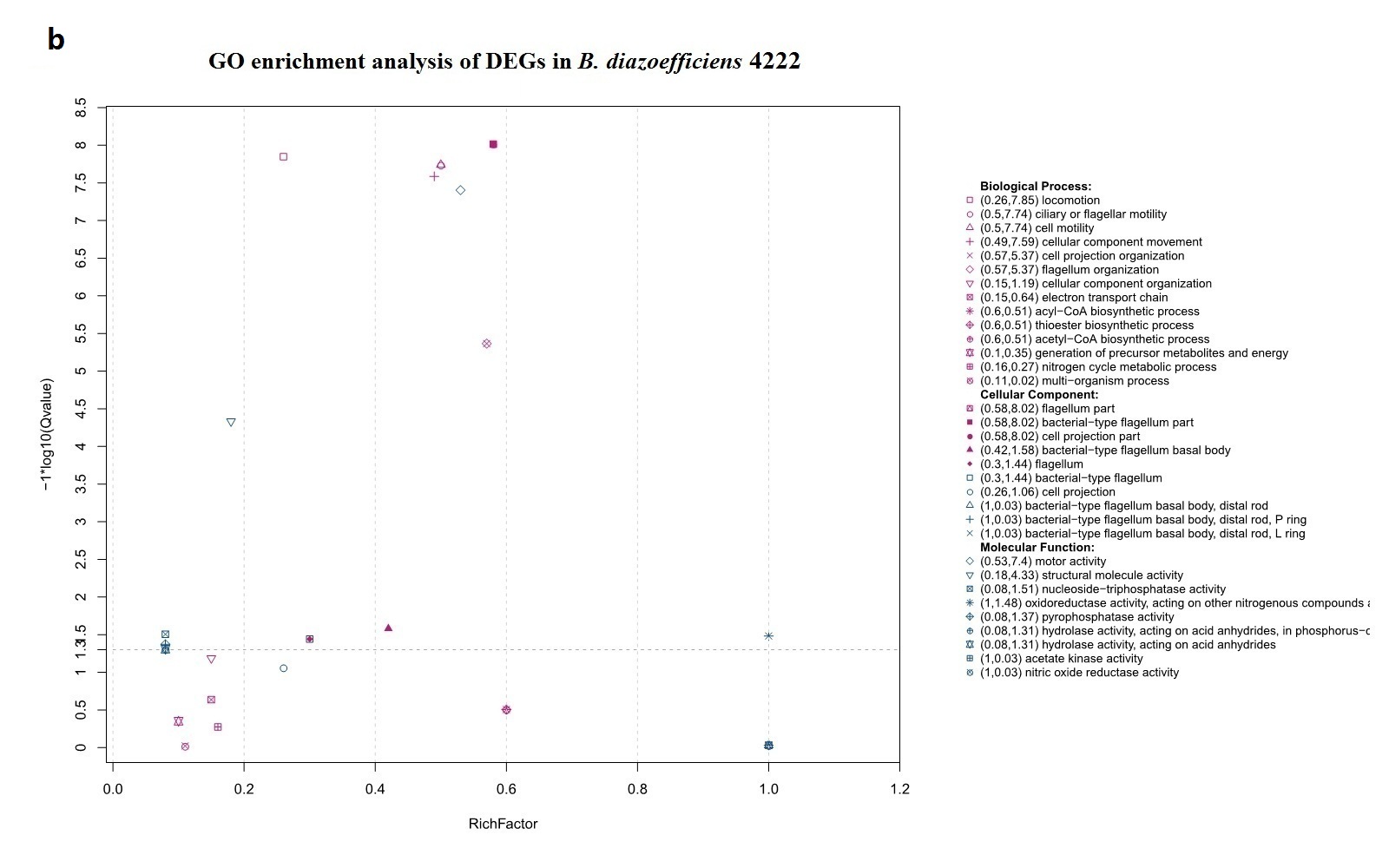
 Fi****gure S4. GO enrichment analysis of the differentially expressed genes in *B. diazoefficiens* 4534(a) and 4222(b), respectively**

The horizontal axis represents rich factor, that is the ratio of the number of DEGs enriched in one GO term to the number of background gene that have identified in this GO functions. while the vertical axis represents enrichment ratio（-log10(q-value), that reflect the statistical significance of enrichment, larger value on coordinate indicates higher GO term relevance. Three subcategories were shown by three different colors。Refer to notation appears on the right side of the diagram to know the icons their corresponding GO term. The dotted lines in parallel with the horizontal axis and vertical axis represent the two screening criteria of enrichment analysis. The dotted in parallel with the horizontal axis represents q=0.05 and the GO term above on the dotted represent significantly enrichment and the dotted in parallel with the vertical axis represents the value of rich factor and a higher value indicates a larger number of DEGs in the GO term.

**
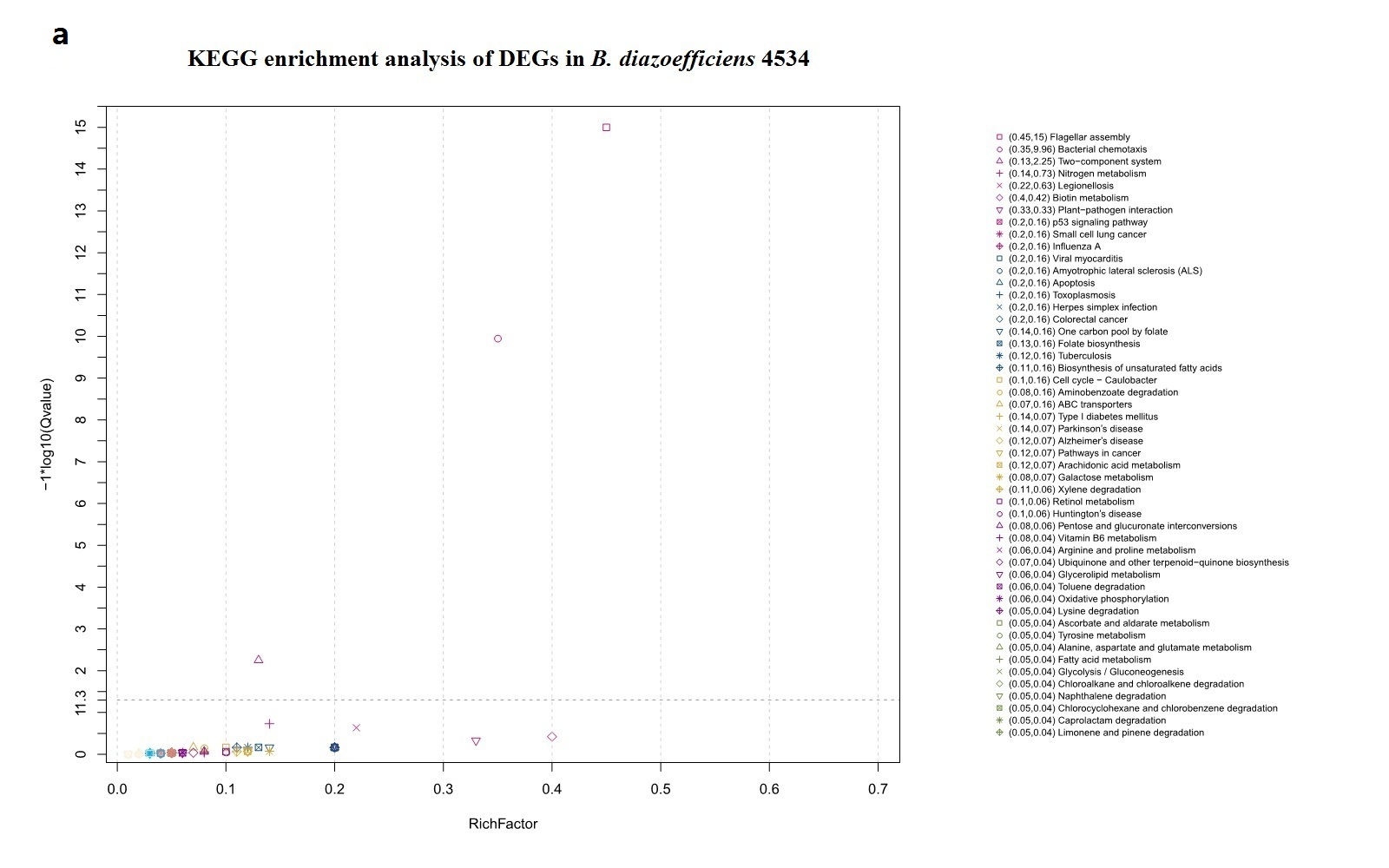
**

**
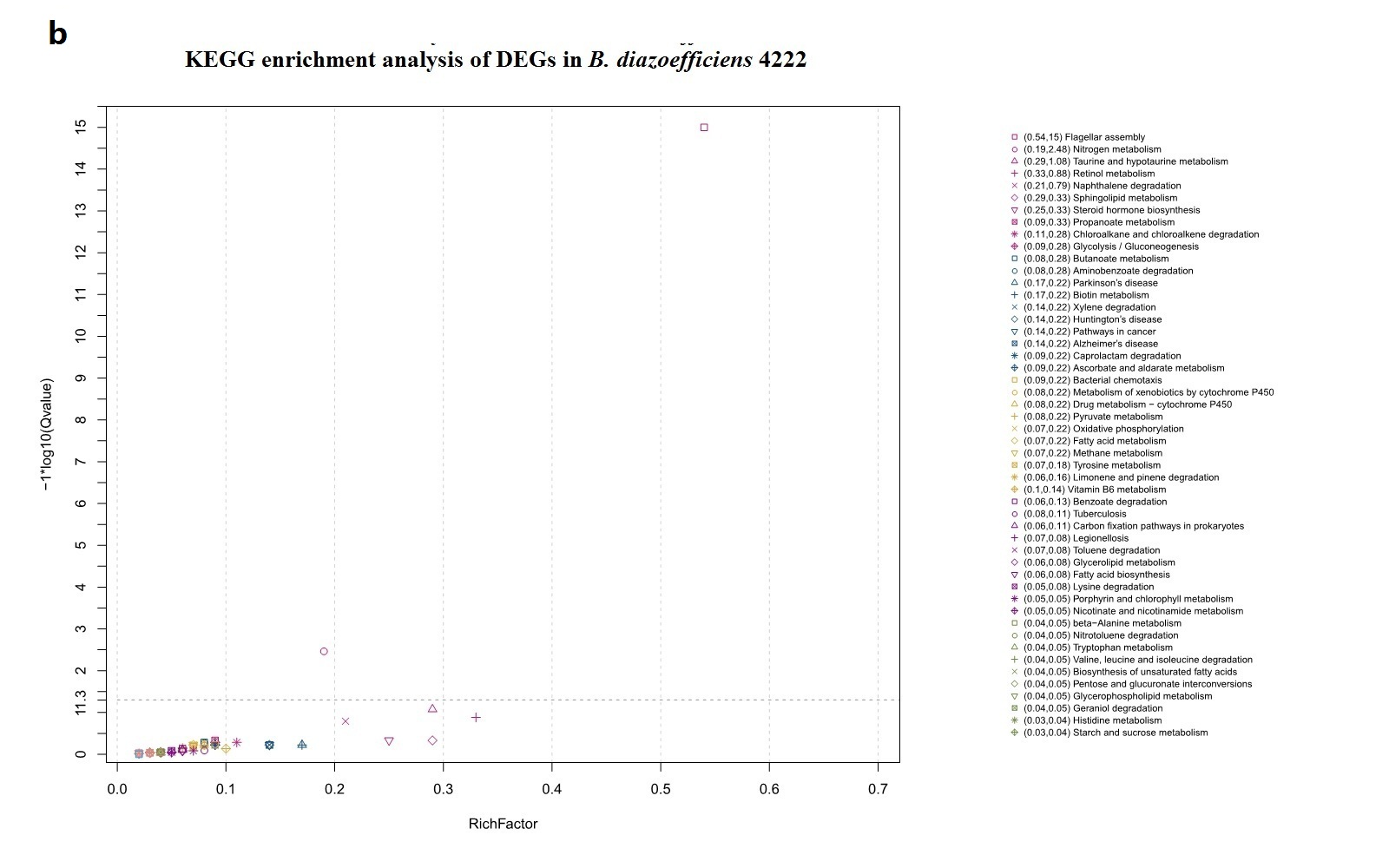
**

**Figure S5. KEEG enrichment analysis of the differentially expressed genes in *B. diazoefficiens* 4534 (b) and 4222(b), respectively.**

The horizontal axis represents rich factor, that is the ratio of the number of DEGs enriched in one KEEG term to the number of background gene that have identified in this KEEG functions. while the vertical axis represents enrichment ratio（-log10(q-value), that reflect the statistical significance of enrichment, larger value on coordinate indicates higher KEEG term relevance. Three subcategories were shown by three different colors。Refer to notation appears on the right side of the diagram to know the icons and their corresponding KEEG term. The dotted lines in parallel with the horizontal axis and vertical axis represent the two screening criteria of enrichment analysis. The dotted in parallel with the horizontal axis represents q=0.05 and the KEEG term above on the dotted represent significantly enrichment and the dotted in parallel with the vertical axis represents the value of rich factor and a higher value indicates a larger number of DEGs in the KEEG term.

**Table S1. The DEGs (differentially expressed genes) were identified in SREs****-4534**

| NO． | Unigene | Locus ID | Log2（folds） | p-value | Description | Up/Down  -regulation |
| --- | --- | --- | --- | --- | --- | --- |
| 1 | unigene00011 | bll2516 | -2.13051165 | 0.001903609 | hypothetical protein bll2516 | down |
| 2 | unigene00074 | bsl257 | -2.262306387 | 0.001413915 | hypothetical protein bsl2574 | down |
| 3 | unigene00091 | —— | -3.614830033 | 1.34E-06 | hypothetical protein S23_53920 | down |
| 4 | unigene00092 | bll2590 | -2.740465372 | 8.49E-05 | hypothetical protein bll2590 | down |
| 5 | unigene00095 | bsl2593 | -2.32732257 | 0.000743679 | hypothetical protein bsl2593 | down |
| 6 | unigene00096 | bsr2594 | -3.078833089 | 1.53E-05 | hypothetical protein bsr2594 | down |
| 7 | unigene00114 | blr2607 | -2.263200568 | 0.001035801 | hypothetical protein blr2607 | down |
| 8 | unigene00119 | blr2611 | -3.94182574 | 9.28E-08 | hypothetical protein blr2611 | down |
| 9 | unigene00144 | bll2636 | -2.605429219 | 0.000552264 | ATP-dependent Clp protease adaptor | down |
| 10 | unigene00175 | bll2664 | -2.394271357 | 0.00157561 | hypothetical protein bll2664 | down |
| 11 | unigene00274 | bll2758 | -3.144971625 | 9.55E-06 | two-component response regulator | down |
| 12 | unigene00277 | blr2761 | -2.137748122 | 0.001723079 | hypothetical protein blr2761 | down |
| 13 | unigene00319 | blr2806 | -3.843896219 | 3.27E-06 | nitrite extrusion protein | down |
| 14 | unigene00333 | bsr2822 | -2.503717661 | 0.00081585 | hypothetical protein bsr2822 | down |
| 15 | unigene00334 | —— | -2.475431348 | 0.002786776 | hypothetical protein blr2823 | down |
| 16 | unigene00347 | blr2836 | -2.325448645 | 0.000756889 | ABC transporter permease | down |
| 17 | unigene00398 | blr2887 | -2.445046339 | 0.000384924 | hypothetical protein blr2887 | down |
| 18 | unigene00534 | bll3023 | -2.195101739 | 0.00141409 | hypothetical protein bll3023 | down |
| 19 | unigene00535 | blr3024 | -2.667640776 | 0.000165641 | hypothetical protein blr3024 | down |
| 20 | unigene00580 | bll3069 | -2.136747558 | 0.001759144 | hypothetical protein bll3069 | down |
| 21 | unigene00584 | bsr3073 | -2.376496333 | 0.00215335 | hypothetical protein bsr3073 | down |
| 22 | unigene00591 | blr3079 | -3.69054848 | 1.30E-06 | quinone oxidoreductase | down |
| 23 | unigene00665 | bll3148 | -3.27212279 | 8.70E-06 | hypothetical protein bll3148 | down |
| 24 | unigene00695 | BBta_3130 | -2.284582062 | 0.001329635 | hypothetical protein S23_48210 | down |
| 25 | unigene00731 | BRADO1773 | -5.083869649 | 1.63E-08 | hypothetical protein bsl3211 | down |
| 26 | unigene00732 | blr3212 | -5.715310928 | 7.59E-12 | nitric oxide reductase subunit E | down |
| 27 | unigene00733 | bsr3213 | -3.670777671 | 0.000211308 | hypothetical protein bsr3213 | down |
| 28 | unigene00734 | blr3214 | -7.724136993 | 1.75E-17 | nitric oxide reductase subunit C | down |
| 29 | unigene00735 | blr3215 | -6.224864905 | 5.15E-14 | nitric oxide reductase subunit B | down |
| 30 | unigene00736 | blr3216 | -5.991161182 | 2.20E-12 | NorQ protein | down |
| 31 | unigene00737 | blr3217 | -4.955886372 | 7.79E-10 | NorD protein | down |
| 32 | unigene00976 | blr3456 | -2.147811847 | 0.001820395 | hypothetical protein blr3456 | down |
| 33 | unigene00982 | bll3462 | -2.484135065 | 0.000869768 | 3-oxoadipate CoA-transferase subunit A | down |
| 34 | unigene00997 | blr3477 | -2.612996391 | 0.000198879 | aspartate aminotransferase | down |
| 35 | unigene01000 | bll3481 | -2.395073489 | 0.000976612 | hypothetical protein bll3481 | down |
| 36 | unigene01001 | bll3482 | -3.400794526 | 0.003042849 | hypothetical protein blr3482 | down |
| 37 | unigene01004 | blr3486 | -2.237889622 | 0.002055378 | hypothetical protein blr3486 | down |
| 38 | unigene01115 | blr7466 | -2.168729447 | 0.001511558 | ribonuclease | down |
| 39 | unigene01154 | —— | -2.850090801 | 0.000148231 | hypothetical protein | down |
| 40 | unigene01155 | —— | -3.368064783 | 8.01E-06 | hypothetical protein | down |
| 41 | unigene01219 | bsl7372 | -2.975349183 | 3.10E-05 | hypothetical protein bsl7372 | down |
| 42 | unigene01306 | blr7290 | -3.256113394 | 4.02E-05 | HpaC | down |
| 43 | unigene01337 | blr7261 | -3.25611184 | 6.55E-06 | Chain A, Crystal Structure Of Bifunctional Proline | down |
| 44 | unigene01384 | bll7217 | -2.124794103 | 0.002122785 | site-specific integrase/recombinase | down |
| 45 | unigene01490 | bsr7112 | -2.394010871 | 0.000637396 | hypothetical protein bsr7112 | down |
| 46 | unigene01491 | bsr7111 | -3.617166603 | 1.25E-06 | hypothetical protein bsr7111 | down |
| 47 | unigene01511 | blr7090 | -3.584321308 | 1.05E-06 | periplasmic nitrate reductase | down |
| 48 | unigene01512 | blr7089 | -4.000850064 | 5.64E-08 | respiratory nitrite reductase | down |
| 49 | unigene01513 | blr7088 | -2.172296056 | 0.001535292 | hypothetical protein blr7088 | down |
| 50 | unigene01514 | bsr7087 | -2.643532742 | 0.000208551 | hypothetical protein bsr7087 | down |
| 51 | unigene01516 | bsl7085 | -6.090771085 | 2.83E-13 | hypothetical protein bsl7085 | down |
| 52 | unigene01518 | bll7083 | -3.196492189 | 7.16E-06 | hypothetical protein bll7083 | down |
| 53 | unigene01520 | bll7081 | -2.633333828 | 0.00087276 | two-component response regulator | down |
| 54 | unigene01542 | blr7060 | -2.268340642 | 0.00122709 | hypothetical protein blr7060 | down |
| 55 | unigene01557 | BBta_1854 | -2.451020303 | 0.000380129 | hypothetical protein BJ6T_23640 | down |
| 56 | unigene01567 | bsr7036 | -2.590703247 | 0.00031451 | periplasmic nitrate reductase | down |
| 57 | unigene01627 | blr6978 | -2.292591809 | 0.00144598 | co-chaperonin GroES | down |
| 58 | unigene01710 | bll6893 | -2.177328288 | 0.001517342 | hypothetical protein bll6893 | down |
| 59 | unigene01929 | —— | -2.246321561 | 0.001287268 | _ | down |
| 60 | unigene01932 | —— | -2.731837432 | 0.000143041 | hypothetical protein | down |
| 61 | unigene01980 | blr6173 | -2.502371222 | 0.000327386 | hypothetical protein blr6173 | down |
| 62 | unigene01991 | blr6182 | -2.616235499 | 0.00018745 | hypothetical protein blr6182 | down |
| 63 | unigene02031 | bll6222 | -3.430103778 | 2.93E-06 | Sec-independent protein translocase | down |
| 64 | unigene02086 | bll6276 | -2.527500765 | 0.000385757 | hypothetical protein bll6276 | down |
| 65 | unigene02349 | bll6540 | -3.563179377 | 2.20E-06 | oxidoreductase | down |
| 66 | unigene02384 | blr6571 | -2.528181478 | 0.00029153 | small heat shock protein | down |
| 67 | unigene02430 | bll6614 | -2.737187209 | 9.35E-05 | esterase | down |
| 68 | unigene02431 | bll6615 | -3.107330714 | 1.14E-05 | hypothetical protein bll6615 | down |
| 69 | unigene02433 | bsl6617 | -2.336870702 | 0.000873668 | hypothetical protein bsl6617 | down |
| 70 | unigene02436 | bll6620 | -2.748610028 | 8.30E-05 | ABC transporter | down |
| 71 | unigene02443 | blr6628 | -3.047157808 | 0.003118634 | hypothetical protein blr6628 | down |
| 72 | unigene02468 | bsl6653 | -2.416959081 | 0.000540563 | hypothetical protein bsl6653 | down |
| 73 | unigene02490 | bll6673 | -2.301381229 | 0.001324441 | hypothetical protein bll6673 | down |
| 74 | unigene02492 | bsl6675 | -3.218678566 | 0.002811985 | hypothetical protein bsl6675 | down |
| 75 | unigene02564 | bsl1006 | -2.454722837 | 0.000477476 | hypothetical protein bsl1006 | down |
| 76 | unigene02634 | blr1072 | -2.734321671 | 0.000132083 | hypothetical protein blr1072 | down |
| 77 | unigene02682 | blr1115 | -2.313232384 | 0.002876578 | transcriptional regulator | down |
| 78 | unigene02786 | —— | -2.823745218 | 6.20E-05 | hypothetical protein S23_08360 | down |
| 79 | unigene02792 | blr1221 | -2.257963626 | 0.001902116 | phosphonate metabolism protein | down |
| 80 | unigene02814 | bll1244 | -2.291247534 | 0.000891186 | flavin dependent oxidoreductase | down |
| 81 | unigene02990 | blr1414 | -2.114189004 | 0.002944952 | transcriptional regulator | down |
| 82 | unigene03106 | blr1527 | -2.526983246 | 0.000562441 | 5-formyltetrahydrofolate cyclo-ligase | down |
| 83 | unigene03131 | blr0850 | -2.490309101 | 0.000325924 | hypothetical protein blr0850 | down |
| 84 | unigene03136 | —— | -2.206327141 | 0.001478454 | hypothetical protein S23_69380 | down |
| 85 | unigene03167 | bll0818 | -2.582441002 | 0.000209427 | hypothetical protein bll0818 | down |
| 86 | unigene03201 | bll0782 | -2.160347444 | 0.001691883 | ribosome-binding factor A | down |
| 87 | unigene03294 | bll0693 | -2.230149513 | 0.001362579 | hypothetical protein bll0693 | down |
| 88 | unigene03326 | bll0661 | -2.031371619 | 0.002783184 | hypothetical protein bll0661 | down |
| 89 | unigene03363 | bll0626 | -2.615377226 | 0.000206913 | hypothetical protein bll0626 | down |
| 90 | unigene03388 | bll0600 | -2.481308661 | 0.000477777 | hypothetical protein bll0600 | down |
| 91 | unigene03411 | bsl0578 | -2.487784976 | 0.000336561 | hypothetical protein bsl0578 | down |
| 92 | unigene03505 | blr0072 | -2.108314276 | 0.002214305 | hypothetical protein blr0072 | down |
| 93 | unigene03508 | bll0076 | 2.587414678 | 0.000525212 | hypothetical protein bll0076 | down |
| 94 | unigene03609 | bll0174 | -2.818503462 | 7.36E-05 | hypothetical protein bll0174 | down |
| 95 | unigene03642 | bsr0210 | -2.091371742 | 0.002265946 | glutaredoxin | down |
| 96 | unigene03689 | bll0256 | -3.009466159 | 5.63E-05 | transcriptional regulator | down |
| 97 | unigene03693 | blr0259 | -3.8000914 | 6.67E-07 | two-component response regulator | down |
| 98 | unigene03707 | bll0271 | -3.823747398 | 0.000191901 | hypothetical protein bll0271 | down |
| 99 | unigene03708 | bll0272 | -5.221815177 | 4.62E-08 | hypothetical protein bll0272 | down |
| 100 | unigene03719 | bll0284 | -3.113129628 | 0.000356572 | malate:quinone oxidoreductase | down |
| 101 | unigene03750 | blr0314 | -2.1928258 | 0.001378415 | nitrous oxide reductase expression regulator | down |
| 102 | unigene03751 | blr0315 | -2.127261132 | 0.001810918 | nitrous-oxide reductase | down |
| 103 | unigene03799 | blr7904 | -2.160656403 | 0.002449221 | DNA-binding transcriptional activator GcvA | down |
| 104 | unigene03807 | blr7897 | -2.581041538 | 0.000227122 | hypothetical protein blr7897 | down |
| 105 | unigene03833 | blr7872 | -2.400538636 | 0.000558067 | HlyD family secretion protein | down |
| 106 | unigene03857 | bsl7850 | -2.757582956 | 8.17E-05 | hypothetical protein bsl7850 | down |
| 107 | unigene03923 | bll7787 | -2.058685467 | 0.002545095 | hypothetical protein bll7787 | down |
| 108 | unigene03929 | bsl7781 | -2.879874396 | 8.45E-05 | hypothetical protein bsl7781 | down |
| 109 | unigene03951 | bsr7757 | -2.293522138 | 0.001097871 | hypothetical protein bsr7757 | down |
| 110 | unigene03969 | blr7740 | -2.883813655 | 3.92E-05 | small heat shock protein | down |
| 111 | unigene04036 | blr4827 | -4.334355692 | 1.36E-08 | hypothetical protein blr4827 | down |
| 112 | unigene04037 | blr4826 | -2.218526707 | 0.001166138 | LexA repressor | down |
| 113 | unigene04044 | bll4820 | -2.364327639 | 0.000581601 | hypothetical protein bll4820 | down |
| 114 | unigene04048 | bll4817 | -2.119535097 | 0.001910404 | hypothetical protein bll4817 | down |
| 115 | unigene04053 | blr4813 | 2.426383045 | 0.000675029 | hypothetical protein blr4813 | down |
| 116 | unigene04081 | bll4785 | -2.152003756 | 0.001706878 | transcriptional regulator | down |
| 117 | unigene04083 | bll4784 | -2.586878949 | 0.000193471 | aldehyde dehydrogenase | down |
| 118 | unigene04089 | —— | 2.902843631 | 0.001433047 | hypothetical protein | down |
| 119 | unigene04145 | bsr4726 | -2.643589365 | 0.000162573 | hypothetical protein bsr4726 | down |
| 120 | unigene04190 | bll4778 | -2.542043019 | 0.000342071 | hypothetical protein | down |
| 121 | unigene04227 | bll4651 | -2.191457714 | 0.001359215 | hypothetical protein bll4651 | down |
| 122 | unigene04351 | bll5323 | -2.174019321 | 0.001448591 | hypothetical protein bll5323 | down |
| 123 | unigene04403 | bsr5273 | -3.741987195 | 3.21E-07 | hypothetical protein bsr5273 | down |
| 124 | unigene04616 | —— | -4.00458104 | 2.03E-07 | hypothetical protein BJ6T_37940 | down |
| 125 | unigene04683 | blr2203 | -2.115628005 | 0.002021015 | RNA polymerase sigma factor | down |
| 126 | unigene04696 | bll2215 | -2.225932429 | 0.002970605 | hypothetical protein bll2215 | down |
| 127 | unigene04701 | —— | -2.247819832 | 0.001584578 | hypothetical protein BJ6T_76290 | down |
| 128 | unigene04714 | blr2231 | -3.287494174 | 1.07E-05 | hypothetical protein blr2231 | down |
| 129 | unigene04715 | blr2231 | -2.869045251 | 4.47E-05 | hypothetical protein blr2231 | down |
| 130 | unigene04771 | blr2285 | -2.379930665 | 0.001016231 | two-component response regulator | down |
| 131 | unigene04919 | bll8082 | -3.029657393 | 0.00065691 | hypothetical protein bll8082 | down |
| 132 | unigene04997 | bll7994 | -2.694843072 | 0.000398695 | hypothetical protein bll7994 | down |
| 133 | unigene04998 | bll7993 | -2.233648706 | 0.001180668 | hypothetical protein bll7993 | down |
| 134 | unigene04999 | bsl7992 | -2.681599671 | 0.000520776 | hypothetical protein bsl7992 | down |
| 135 | unigene05009 | bll7982 | -2.347450572 | 0.000635793 | hypothetical protein bll7982 | down |
| 136 | unigene05030 | blr7961 | -2.604685944 | 0.000191425 | HspC2 heat shock protein | down |
| 137 | unigene05031 | bll7960 | -2.681541311 | 0.000503071 | hypothetical protein bll7960 | down |
| 138 | unigene05197 | blr5150 | -2.107361812 | 0.00212042 | hypothetical protein blr5150 | down |
| 139 | unigene05213 | bsl5165 | -2.462662166 | 0.000403623 | hypothetical protein bsl5165 | down |
| 140 | unigene05262 | blr4582 | -2.454983582 | 0.000487269 | acetylornithine transaminase | down |
| 141 | unigene05418 | bll3835 | -2.91992427 | 3.80E-05 | hypothetical protein bll3835 | down |
| 142 | unigene05474 | blr3787 | -2.761812405 | 0.000152934 | hypothetical protein blr3787 | down |
| 143 | unigene05585 | blr4433 | -2.914495528 | 7.58E-05 | hypothetical protein blr4433 | down |
| 144 | unigene05645 | bll4381 | -2.422125168 | 0.001301833 | transcriptional regulator | down |
| 145 | unigene05832 | bll4873 | -2.055265295 | 0.002607268 | hypothetical protein bll4873 | down |
| 146 | unigene05841 | bll4882 | -3.234449389 | 1.07E-05 | hypothetical protein bll4882 | down |
| 147 | unigene05961 | bll4998 | -2.740637101 | 0.000155322 | hypothetical protein bll4998 | down |
| 148 | unigene05991 | blr0422 | -2.034564333 | 0.002841377 | hypothetical protein blr0422 | down |
| 149 | unigene06053 | bll0484 | -2.492137309 | 0.001711022 | transcriptional regulator | down |
| 150 | unigene06061 | blr0492 | -3.063778505 | 0.000110084 | hypothetical protein blr0492 | down |
| 151 | unigene06066 | blr0497 | -3.054125046 | 1.54E-05 | hypothetical protein blr0497 | down |
| 152 | unigene06099 | blr4013 | -2.434642588 | 0.000732926 | transcriptional regulator | down |
| 153 | unigene06152 | blr3963 | -2.146157691 | 0.002117467 | transcriptional regulator | down |
| 154 | unigene06207 | bll3909 | -2.411288955 | 0.000514513 | hypothetical protein bll3909 | down |
| 155 | unigene06234 | —— | -2.052117166 | 0.002509789 | Sulfur globule protein precursor | down |
| 156 | unigene06246 | blr4131 | -2.667842358 | 0.000141408 | hypothetical protein blr4131 | down |
| 157 | unigene06265 | bll4149 | -2.239438308 | 0.001205925 | glutathione peroxidase | down |
| 158 | unigene06281 | blr4162 | -2.541967425 | 0.000249391 | hypothetical protein blr4162 | down |
| 159 | unigene06282 | —— | -2.362398794 | 0.00058987 | hypothetical protein S23_40940 | down |
| 160 | unigene06292 | blr4174 | -2.55376709 | 0.000299343 | hypothetical protein blr4174 | down |
| 161 | unigene06329 | bll4208 | -2.561656656 | 0.000614539 | hypothetical protein bll4208 | down |
| 162 | unigene06331 | blr4210 | -2.516394355 | 0.000322558 | hypothetical protein blr4210 | down |
| 163 | unigene06343 | bll4221 | -2.344407069 | 0.001004178 | transcriptional regulator | down |
| 164 | unigene06363 | blr5735 | -2.805097819 | 9.01E-05 | transcriptional regulator | down |
| 165 | unigene06390 | —— | -2.201247729 | 0.002844046 | hypothetical protein | down |
| 166 | unigene06403 | bll5772 | -2.82545348 | 6.48E-05 | hypothetical protein bll5772 | down |
| 167 | unigene06405 | blr5774 | -2.170410251 | 0.001623981 | sulfide-quinone reductase | down |
| 168 | unigene06409 | blr5778 | -2.162943328 | 0.001881807 | nitrogen fixation protein | down |
| 169 | unigene06562 | blr4241 | -2.136823712 | 0.001985984 | hypothetical protein blr4241 | down |
| 170 | unigene06563 | blr4240 | -2.308775593 | 0.000802249 | hypothetical protein blr4240 | down |
| 171 | unigene06569 | bsr4236 | -3.021901092 | 2.08E-05 | hypothetical protein bsr4236 | down |
| 172 | unigene06626 | bll0933 | -2.322502032 | 0.000752301 | transcriptional regulator | down |
| 173 | unigene06663 | bll2388 | -2.505086715 | 0.000426507 | cytochrome c2 | down |
| 174 | unigene06716 | bsl2435 | -2.40676359 | 0.000486564 | hypothetical protein bsl2435 | down |
| 175 | unigene06737 | blr2455 | -3.196737719 | 7.29E-06 | isocitrate lyase | down |
| 176 | unigene06738 | RPC_1380 | -2.750888223 | 0.000354528 | hypothetical protein BJ6T_73800 | down |
| 177 | unigene06759 | bll2471 | -2.583677067 | 0.000317297 | hypothetical protein bll2471 | down |
| 178 | unigene06845 | blr5625 | -2.452879661 | 0.000402578 | co-chaperonin GroES | down |
| 179 | unigene06883 | blr7629 | -5.008441218 | 1.27E-09 | hypothetical protein blr7629 | down |
| 180 | unigene06884 | bll7628 | -7.009403664 | 6.55E-14 | hypothetical protein bll7628 | down |
| 181 | unigene06885 | bll7627 | -5.306392015 | 3.89E-10 | hypothetical protein bll7627 | down |
| 182 | unigene06886 | bll7626 | -3.370037042 | 2.32E-05 | hypothetical protein bll7626 | down |
| 183 | unigene06889 | blr7623 | -2.463313375 | 0.000555396 | hypothetical protein blr7623 | down |
| 184 | unigene06921 | blr7595 | -5.168573096 | 4.04E-11 | hypothetical protein blr7595 | down |
| 185 | unigene06922 | blr7594 | -4.973047527 | 1.30E-10 | multidrug resistance protein | down |
| 186 | unigene06923 | blr7593 | -3.808467631 | 1.83E-07 | multidrug resistance efflux pump | down |
| 187 | unigene06955 | bll4072 | -2.278968045 | 0.000947139 | replicative DNA helicase | down |
| 188 | unigene07032 | —— | -2.134462113 | 0.001796607 | hypothetical protein | down |
| 189 | unigene07033 | blr0369 | -2.818916667 | 5.91E-05 | hypothetical protein blr0369 | down |
| 190 | unigene07037 | blr0366 | -2.102408601 | 0.002028199 | hypothetical protein blr0366 | down |
| 191 | unigene07092 | —— | -2.926756377 | 0.00011347 | _ | down |
| 192 | unigene07160 | bll5689 | -3.178935597 | 7.61E-06 | transcriptional regulator | down |
| 193 | unigene07190 | bll5661 | -2.017973009 | 0.003087834 | hypothetical protein bll5661 | down |
| 194 | unigene07196 | bll5655 | -2.252674796 | 0.000989354 | alcohol dehydrogenase | down |
| 195 | unigene07227 | —— | -2.035581627 | 0.00280032 | molecular chaperone GroEL | down |
| 196 | unigene07295 | bll8143 | -2.482791451 | 0.000409766 | hypothetical protein bll8143 | down |
| 197 | unigene07345 | bll6069 | -2.343222667 | 0.000644502 | hypothetical protein bll6069 | down |
| 198 | unigene07347 | blr6067 | -2.108215341 | 0.002021206 | hypothetical protein blr6067 | down |
| 199 | unigene07348 | bsr6066 | -2.672972991 | 0.000128575 | hypothetical protein bsr6066 | down |
| 200 | unigene07352 | —— | -2.470650374 | 0.000371083 | cytochrome C6 | down |
| 201 | unigene07412 | blr7585 | -2.990335862 | 0.000755559 | hypothetical protein blr7585 | down |
| 202 | unigene07882 | blr1670 | -2.137034611 | 0.001730345 | hypothetical protein blr1670 | down |
| 203 | unigene08006 | bll4645 | -2.201745117 | 0.00136098 | hypothetical protein bll4645 | down |
| 204 | unigene08007 | bll4644 | -2.007699286 | 0.003154406 | hypothetical protein bll4644 | down |
| 205 | unigene00047 | blr2548 | 2.111788457 | 0.002262826 | chemotaxis protein | up |
| 206 | unigene00061 | bll2562 | 2.586342879 | 0.002278477 | GDP-mannose mannosyl hydrolase | up |
| 207 | unigene00183 | bll2674 | 2.776178597 | 9.39E-05 | sugar ABC transporter substrate-binding protein | up |
| 208 | unigene00188 | bll2679 | 3.231310201 | 0.000192859 | dioxygenase | up |
| 209 | unigene00195 | blr2686 | 5.49883102 | 1.73E-11 | hypothetical protein blr2686 | up |
| 210 | unigene00196 | blr2687 | 7.911650233 | 7.00E-16 | metabolite transport protein | up |
| 211 | unigene00197 | blr2688 | 8.228366875 | 4.35E-17 | vanillin: NAD oxidoreductase | up |
| 212 | unigene00198 | blr2689 | 5.84217264 | 7.29E-12 | benzoylformate decarboxylase | up |
| 213 | unigene00199 | blr2690 | 4.051175081 | 0.00011434 | benzoylformate decarboxylase | up |
| 214 | unigene00378 | bll2868 | 2.576746093 | 0.000523713 | ABC transporter substrate-binding protein | up |
| 215 | unigene00428 | bll2917 | 2.102766531 | 0.002515042 | hypothetical protein bll2917 | up |
| 216 | unigene00432 | blr2921 | 3.150308575 | 1.18E-05 | hypothetical protein blr2921 | up |
| 217 | unigene00433 | blr2922 | 4.24583651 | 1.49E-08 | amino acid ABC transporter substrate-binding protein | up |
| 218 | unigene00434 | blr2923 | 5.339809642 | 2.73E-10 | amino acid ABC transporter permease | up |
| 219 | unigene00435 | blr2934 | 5.618774636 | 3.16E-11 | amino acid ABC transporter permease | up |
| 220 | unigene00436 | blr2925 | 6.285410414 | 1.28E-12 | amino acid ABC transporter ATP-binding protein | up |
| 221 | unigene00437 | blr2926 | 5.069266183 | 5.85E-10 | amino acid ABC transporter ATP-binding protein | up |
| 222 | unigene00442 | blr2931 | 3.006625318 | 2.43E-05 | methyl-accepting chemotaxis protein | up |
| 223 | unigene00560 | bll3049 | 2.131108634 | 0.003166673 | hypothetical protein bll3049 | up |
| 224 | unigene00623 | bll3111 | 2.520010881 | 0.000564561 | hypothetical protein bll3111 | up |
| 225 | unigene00624 | bll3112 | 2.352735473 | 0.000851479 | glucosyl transferase | up |
| 226 | unigene00720 | blr3200 | 4.818456394 | 4.22E-10 | sugar ABC transporter substrate-binding protein | up |
| 227 | unigene00721 | blr3201 | 4.493507582 | 7.56E-09 | sugar ABC transporter ATP-binding protein | up |
| 228 | unigene00722 | blr3202 | 5.500149299 | 3.98E-10 | sugar ABC transporter permease | up |
| 229 | unigene00723 | blr3203 | 3.390882532 | 4.28E-06 | ABC transporter permease | up |
| 230 | unigene00724 | blr3204 | 2.27434591 | 0.001121416 | transcriptional regulator | up |
| 231 | unigene00725 | blr3205 | 4.427795523 | 1.30E-08 | dehydrogenase | up |
| 232 | unigene00726 | blr3206 | 3.569458444 | 1.14E-06 | aldose 1-epimerase | up |
| 233 | unigene00727 | blr3207 | 2.84413543 | 6.78E-05 | hypothetical protein blr3207 | up |
| 234 | unigene00728 | blr3208 | 4.53455038 | 2.12E-09 | sugar ABC transporter substrate-binding protein | up |
| 235 | unigene00729 | blr3209 | 3.839929594 | 2.02E-07 | sugar ABC transporter ATP-binding protein | up |
| 236 | unigene00730 | blr3210 | 3.77320504 | 4.09E-07 | sugar ABC transporter permease | up |
| 237 | unigene00881 | bll3360 | 2.466621408 | 0.000951917 | hypothetical protein bll3360 | up |
| 238 | unigene00887 | blr3366 | 2.35236628 | 0.001095298 | hypothetical protein blr3366 | up |
| 239 | unigene00888 | blr3367 | 3.678528948 | 1.27E-05 | cellulase | up |
| 240 | unigene00960 | bll3440 | 2.886001905 | 0.000592004 | hypothetical protein bll3440 | up |
| 241 | unigene00965 | blr3445 | 2.21555173 | 0.001419976 | enoyl-CoA hydratase | up |
| 242 | unigene00966 | blr3446 | 2.416639696 | 0.000604695 | 6-carboxyhexanoate--CoA ligase | up |
| 243 | unigene00967 | blr3447 | 2.815885386 | 9.59E-05 | substrate-binding protein | up |
| 244 | unigene00968 | blr3448 | 2.75686968 | 0.000187569 | acyl-CoA dehydrogenase | up |
| 245 | unigene00969 | blr3449 | 3.188147543 | 6.98E-05 | dehydrogenase | up |
| 246 | unigene00971 | blr3451 | 2.591348853 | 0.00040743 | oxidoreductase | up |
| 247 | unigene00972 | blr3452 | 2.871321129 | 0.000146123 | hypothetical protein blr3452 | up |
| 248 | unigene00973 | blr3453 | 2.780787307 | 0.000974726 | hypothetical protein blr3453 | up |
| 249 | unigene00974 | blr3454 | 2.934539417 | 9.57E-05 | hypothetical protein blr3454 | up |
| 250 | unigene01067 | blr7520 | 2.976328236 | 0.002762764 | hypothetical protein blr7520 | up |
| 251 | unigene01091 | blr7491 | 2.283662474 | 0.001100579 | 2-keto-gluconate dehydrogenase | up |
| 252 | unigene01093 | blr7489 | 3.232585769 | 0.000356572 | lactoylglutathione lyase | up |
| 253 | unigene01183 | bll7406 | 2.633748841 | 0.000153877 | hypothetical protein bll7406 | up |
| 254 | unigene01184 | bll7405 | 2.654813338 | 0.000176242 | hypothetical protein bll7405 | up |
| 255 | unigene01292 | bll7303 | 2.506780029 | 0.001527531 | hypothetical protein bll7303 | up |
| 256 | unigene01370 | bll7229 | 2.539606898 | 0.000477594 | hypothetical protein bll7229 | up |
| 257 | unigene01460 | blr7142 | 2.14398767 | 0.003165856 | hypothetical protein blr7142 | up |
| 258 | unigene01461 | bsl7141 | 2.937474239 | 3.95E-05 | components of type IV pilus pilin subunit | up |
| 259 | unigene01526 | bll7076 | 2.244428621 | 0.001027439 | HmuR protein | up |
| 260 | unigene01528 | bll7074 | 2.213684519 | 0.001260566 | hypothetical protein bll7074 | up |
| 261 | unigene01612 | blr6997 | 4.713913401 | 1.43E-08 | flagellar hook assembly protein | up |
| 262 | unigene01613 | blr6996 | 4.89414465 | 4.12E-09 | hypothetical protein blr6996 | up |
| 263 | unigene01645 | bll6959 | 2.196759836 | 0.002314444 | hydrolase | up |
| 264 | unigene01718 | blr6883 | 3.231310201 | 0.000192859 | hypothetical protein blr6883 | up |
| 265 | unigene01720 | bll6881 | 3.322857979 | 0.000469533 | hypothetical protein bll6881 | up |
| 266 | unigene01723 | bll6878 | 2.572905035 | 0.001516278 | flagellar motor switch protein | up |
| 267 | unigene01724 | bll6877 | 4.397429348 | 0.000408153 | flagellar biosynthesis protein FlhB | up |
| 268 | unigene01725 | bll6876 | 3.533488383 | 0.00025588 | flagellar basal-body rod protein FlgB | up |
| 269 | unigene01726 | bll6875 | 7.683161868 | 5.43E-07 | flagellar basal body rod protein FlgC | up |
| 270 | unigene01728 | bll6873 | 3.511487152 | 4.71E-05 | flagellar basal body rod protein FlgG | up |
| 271 | unigene01729 | bll6872 | 4.361186137 | 0.000497338 | flagellar basal body P-ring biosynthesis protein FlgA | up |
| 272 | unigene01730 | bll6871 | 2.925814029 | 0.001433047 | flagellar basal body P-ring protein | up |
| 273 | unigene01735 | bll6866 | 2.429321492 | 0.000901749 | flagellin | up |
| 274 | unigene01736 | bll6865 | 2.342993917 | 0.000947994 | flagellin | up |
| 275 | unigene01737 | bll6864 | 3.229146157 | 8.66E-05 | flagellar MS-ring protein | up |
| 276 | unigene01738 | bll6863 | 3.749565601 | 0.000405979 | hypothetical protein bll6863 | up |
| 277 | unigene01739 | bll6862 | 2.780351137 | 0.001042881 | flagellar motor protein MotB | up |
| 278 | unigene01741 | bll6860 | 3.778950393 | 7.67E-05 | hypothetical protein bll6860 | up |
| 279 | unigene01743 | bll6858 | 4.08811362 | 2.82E-07 | flagellar hook protein FlgE | up |
| 280 | unigene01744 | bll6857 | 3.395309863 | 5.42E-05 | flagellar hook-associated protein FlgK | up |
| 281 | unigene01745 | bll6856 | 3.87489883 | 7.05E-06 | flagellar hook-associated protein FlgL | up |
| 282 | unigene01746 | bll6855 | 6.95842024 | 1.80E-05 | flagellar biosynthesis regulatory protein FlaF | up |
| 283 | unigene01748 | bll6853 | 7.322595996 | 3.54E-06 | flagellar basal body rod modification protein | up |
| 284 | unigene01750 | bll6851 | 3.356107059 | 0.000189466 | flagellar biosynthesis protein FlhA | up |
| 285 | unigene01751 | bll6850 | 6.042823785 | 0.001006409 | flagellar biosynthesis protein FliR | up |
| 286 | unigene01754 | bll6848 | 6.521984791 | 0.000125561 | hypothetical protein bll6848 | up |
| 287 | unigene01755 | bll6847 | 6.112170697 | 0.000726851 | hypothetical protein bll6847 | up |
| 288 | unigene01767 | bll6834 | 3.845141963 | 4.48E-07 | ABC transporter substrate-binding protein | up |
| 289 | unigene01768 | bll6833 | 3.422215408 | 0.000140031 | ABC transporter permease | up |
| 290 | unigene01769 | bll6832 | 2.997494244 | 9.92E-05 | ABC transporter permease | up |
| 291 | unigene01770 | bll6831 | 2.704741681 | 0.000361494 | L-idonate 5-dehydrogenase | up |
| 292 | unigene01771 | bll6830 | 2.619939815 | 0.000618074 | mannonate dehydratase | up |
| 293 | unigene01797 | blr6804 | 2.788788489 | 9.21E-05 | substrate-binding protein | up |
| 294 | unigene01862 | blr6743 | 2.151502509 | 0.002667924 | ferredoxin oxidoreductase subunit alpha | up |
| 295 | unigene01866 | blr6740 | 2.336185799 | 0.001069552 | proline iminopeptidase | up |
| 296 | unigene02045 | bll6235 | 6.95842024 | 1.80E-05 | ABC transporter permease | up |
| 297 | unigene02046 | bll6236 | 4.361804449 | 3.68E-06 | ABC transporter substrate-binding protein | up |
| 298 | unigene02058 | blr6248 | 2.826547456 | 0.000268124 | ABC transporter ATP-binding protein | up |
| 299 | unigene02155 | bll6350 | 2.766831937 | 0.000633856 | tagatose 1,6-diphosphate aldolase | up |
| 300 | unigene02208 | bll6403 | 5.89325007 | 0.001408973 | ABC transporter permease | up |
| 301 | unigene02212 | bll6407 | 2.827498105 | 0.00087276 | ABC transporter substrate-binding protein | up |
| 302 | unigene02301 | bll6496 | 2.168748824 | 0.001843503 | hypothetical protein bll6496 | up |
| 303 | unigene02317 | bll6510 | 2.279517653 | 0.002559234 | dihydrofolate reductase | up |
| 304 | unigene02424 | bll6608 | 2.033791384 | 0.002980883 | penicillin binding protein 2 | up |
| 305 | unigene02441 | bll6625 | 2.121518143 | 0.002278179 | hypothetical protein bll6625 | up |
| 306 | unigene02613 | blr1052 | 2.489484001 | 0.000350681 | ABC transporter binding protein | up |
| 307 | unigene02627 | blr1065 | 2.627954897 | 0.000333837 | ABC transporter permease | up |
| 308 | unigene02628 | blr1066 | 2.832054294 | 0.000416033 | ABC transporter ATP-binding protein | up |
| 309 | unigene02629 | blr1067 | 2.946648784 | 0.000145045 | ABC transporter ATP-binding protein | up |
| 310 | unigene02638 | bll1076 | 2.830654656 | 7.41E-05 | anaerobic benzoate catabolism transcriptional regulator | up |
| 311 | unigene02640 | blr1077 | 2.577523246 | 0.00028586 | acetyl-CoA synthetase | up |
| 312 | unigene02641 | blr1078 | 2.820975568 | 0.000168354 | hydrolase | up |
| 313 | unigene02643 | blr1080 | 4.842326545 | 3.33E-10 | benzoyl-CoA-dihydrodiol lyase | up |
| 314 | unigene02644 | blr108 | 5.400616068 | 8.56E-12 | benzoyl-CoA oxygenase component | up |
| 315 | unigene02646 | bll1083 | 2.186317002 | 0.001646088 | hypothetical protein bll1083 | up |
| 316 | unigene02723 | bll1155 | 2.06949434 | 0.002451911 | hypothetical protein bll1155 | up |
| 317 | unigene02741 | bsr1173 | 2.85177592 | 0.000720674 | CoxF protein | up |
| 318 | unigene02870 | blr1303 | 3.079895503 | 0.001947577 | hypothetical protein blr1303 | up |
| 319 | unigene02872 | bll1305 | 2.156973205 | 0.002124177 | hypothetical protein bll1305 | up |
| 320 | unigene03012 | bll1434 | 2.851592537 | 7.38E-05 | pilus assemble protein | up |
| 321 | unigene03013 | bll1435 | 2.646108435 | 0.000200622 | pilus assembly protein | up |
| 322 | unigene03014 | bll1436 | 2.250846505 | 0.001208317 | pilus assembly protein | up |
| 323 | unigene03015 | bll1437 | 2.341564914 | 0.00075327 | pilus assembly protein | up |
| 324 | unigene03016 | bll1438 | 2.249762707 | 0.001464635 | pilus assembly protein | up |
| 325 | unigene03762 | bll0326 | 2.604028091 | 0.000250318 | methyl-accepting chemotaxis protein | up |
| 326 | unigene03764 | bll7938 | 2.180810344 | 0.001575356 | hypothetical protein bll7938 | up |
| 327 | unigene03768 | blr7934 | 2.689499766 | 0.0003969 | hypothetical protein blr7934 | up |
| 328 | unigene03912 | blr7797 | 2.604028 | 0.000250318 | RNA polymerase sigma factor σ^EcfG^ | up |
| 329 | unigene03913 | bll7795 | 3.743454 | 0.00013463 | two-component response regulator *phyR* | up |
| 330 | unigene03984 | blr7722 | 6.042823785 | 0.001006409 | hypothetical protein blr7722 | up |
| 331 | unigene04016 | blr4846 | 2.9591118 | 0.002266129 | oxidoreductase | up |
| 332 | unigene04237 | blr5436 | 2.691930925 | 0.001117054 | hypothetical protein blr5436 | up |
| 333 | unigene04238 | blr5435 | 2.74239521 | 0.000173057 | hypothetical protein blr5435 | up |
| 334 | unigene04239 | blr5434 | 2.355011869 | 0.000832234 | D-mycarose 3-C-methyltransferase | up |
| 335 | unigene04242 | blr5431 | 2.560177788 | 0.00101181 | hypothetical protein blr5431 | up |
| 336 | unigene04244 | blr5429 | 2.592396389 | 0.000351091 | hypothetical protein blr5429 | up |
| 337 | unigene04245 | blr5428 | 2.365795159 | 0.000750454 | hypothetical protein blr5428 | up |
| 338 | unigene04422 | bll5254 | 2.988908295 | 0.000189598 | hypothetical protein bll5254 | up |
| 339 | unigene04527 | blr6005 | 2.584270855 | 0.000649513 | hypothetical protein blr6005 | up |
| 340 | unigene04529 | blr6002 | 2.337624184 | 0.000871911 | hypothetical protein blr6002 | up |
| 341 | unigene04530 | blr6003 | 2.504891968 | 0.000673408 | glycosyl transferase family protein | up |
| 342 | unigene04549 | bll5982 | 2.633433539 | 0.000240723 | hypothetical protein bll5982 | up |
| 343 | unigene04673 | blr2194 | 2.432271918 | 0.000654037 | chemotaxis two-component response regulator | up |
| 344 | unigene04674 | blr2195 | 2.43448551 | 0.000552986 | chemotaxis-specific methylesterase | up |
| 345 | unigene04675 | blr2196 | 2.743453828 | 0.00013463 | protein-glutamate O-methyltransferase | up |
| 346 | unigene04677 | bll2198 | 2.470348863 | 0.001750181 | hypothetical protein bll2198 | up |
| 347 | unigene04733 | blr2247 | 2.530566215 | 0.001335432 | fatty acid desaturase | up |
| 348 | unigene04758 | blr2271 | 3.169972946 | 0.000170349 | sugar ABC transporter permease | up |
| 349 | unigene04759 | blr2272 | 2.979817343 | 4.14E-05 | porin | up |
| 350 | unigene04828 | blr2343 | 2.328492338 | 0.000778398 | chemotaxis protein CheA | up |
| 351 | unigene04829 | blr2344 | 3.246693662 | 2.60E-05 | chemotaxis protein | up |
| 352 | unigene04830 | blr2345 | 2.803495183 | 7.24E-05 | methyl-accepting chemotaxis protein | up |
| 353 | unigene04831 | blr2346 | 2.826547456 | 0.000268124 | chemotaxis protein | up |
| 354 | unigene04832 | blr2347 | 3.162221407 | 1.12E-05 | sensory transducer protein | up |
| 355 | unigene04833 | blr2348 | 4.255501926 | 1.30E-07 | protein-glutamate O-methyltransferase | up |
| 356 | unigene04835 | blr2350 | 3.436060273 | 9.03E-06 | hypothetical protein blr2350 | up |
| 357 | unigene04859 | blr2369 | 2.272494289 | 0.00206738 | exopolysaccharide production protein | up |
| 358 | unigene05037 | bll7954 | 2.035915451 | 0.003032327 | methyl accepting chemotaxis protein | up |
| 359 | unigene05054 | bll5012 | 2.24045215 | 0.001412104 | type I restriction enzyme | up |
| 360 | unigene05055 | bll5013 | 2.091388274 | 0.002961104 | hypothetical protein bll5013 | up |
| 361 | unigene05127 | bll5085 | 2.706928059 | 0.000107114 | hypothetical protein bll5085 | up |
| 362 | unigene05276 | blr4568 | 3.239807286 | 7.66E-06 | LamB/YcsF family protein | up |
| 363 | unigene05277 | blr4567 | 2.741354968 | 0.000105811 | hypothetical protein blr4567 | up |
| 364 | unigene05278 | blr4566 | 2.360558802 | 0.000729861 | hypothetical protein blr4566 | up |
| 365 | unigene05280 | bll4564 | 2.358446714 | 0.000776046 | hypothetical protein | up |
| 366 | unigene05459 | blr3801 | 2.220125831 | 0.001626843 | chemotaxis protein | up |
| 367 | unigene05529 | bll3736 | 2.278989269 | 0.001370657 | hypothetical protein bll3736 | up |
| 368 | unigene05651 | bll4376 | 2.781423132 | 0.000156505 | hypothetical protein bll4376 | up |
| 369 | unigene05652 | blr4375 | 2.542313842 | 0.00124941 | hypothetical protein blr4375 | up |
| 370 | unigene05653 | blr4374 | 4.818522182 | 4.06E-09 | 4-hydroxythreonine-4-phosphate dehydrogenase | up |
| 371 | unigene05654 | blr4373 | 4.241318529 | 2.25E-07 | hypothetical protein blr4373 | up |
| 372 | unigene05655 | blr4372 | 2.888055723 | 0.000671796 | hypothetical protein blr4372 | up |
| 373 | unigene05656 | blr4371 | 3.880799722 | 3.64E-07 | hypothetical protein blr4371 | up |
| 374 | unigene05657 | blr4370 | 3.479611241 | 3.13E-06 | hypothetical protein blr4370 | up |
| 375 | unigene05658 | blr4369 | 2.534450305 | 0.000431793 | enoyl-CoA hydratase | up |
| 376 | unigene05813 | blr3599 | 2.651310669 | 0.00054842 | hypothetical protein blr3599 | up |
| 377 | unigene05823 | bll3588 | 2.781364158 | 0.001544939 | hypothetical protein bll3588 | up |
| 378 | unigene05863 | bll4903 | 2.231556828 | 0.002762034 | biotin--protein ligase | up |
| 379 | unigene05864 | bll4904 | 2.206158438 | 0.00152307 | NADH dehydrogenase subunit N | up |
| 380 | unigene05865 | bll4905 | 2.172523066 | 0.001610684 | NADH dehydrogenase subunit M | up |
| 381 | unigene05866 | bll4906 | 2.251410649 | 0.001097762 | NADH dehydrogenase subunit L | up |
| 382 | unigene05868 | bll4908 | 2.602868948 | 0.000435015 | NADH dehydrogenase subunit J | up |
| 383 | unigene05897 | blr4936 | 2.413747748 | 0.000711716 | cation efflux system protein | up |
| 384 | unigene05898 | blr4937 | 2.562674001 | 0.000256217 | cation efflux system protein | up |
| 385 | unigene05934 | blr4971 | 2.711520375 | 0.000253507 | hypothetical protein blr4971 | up |
| 386 | unigene05935 | bll4972 | 2.035752449 | 0.003103737 | succinoglycan transport protein | up |
| 387 | unigene05936 | blr4973 | 2.833366597 | 0.000263768 | glycosyl transferase family protein | up |
| 388 | unigene05937 | blr4974 | 2.193163165 | 0.002107283 | polysaccharide biosynthesis glycosyltransferase | up |
| 389 | unigene05938 | blr4975 | 2.960344337 | 0.000478524 | hypothetical protein blr4975 | up |
| 390 | unigene06052 | blr0483 | 2.330260756 | 0.000826428 | 16S rRNA-processing protein RimM | up |
| 391 | unigene06171 | blr3943 | 2.325334023 | 0.001277488 | hypothetical protein blr3943 | up |
| 392 | unigene06173 | blr3942 | 2.599658551 | 0.000961122 | hypothetical protein blr3942 | up |
| 393 | unigene06174 | blr3941 | 2.137686613 | 0.002014798 | hypothetical protein blr3941 | up |
| 394 | unigene06195 | blr3920 | 2.130050693 | 0.002413997 | ABC transporter permease | up |
| 395 | unigene06196 | blr3919 | 2.238488566 | 0.001211751 | ABC transporter permease | up |
| 396 | unigene06197 | blr3918 | 2.137605541 | 0.001734813 | ABC transporter substrate-binding protein | up |
| 397 | unigene06203 | bll3913 | 2.269317791 | 0.001323961 | 3-oxoacyl-ACP reductase | up |
| 398 | unigene06459 | blr5827 | 4.073480732 | 1.39E-06 | flagellar basal body rod protein FlgF | up |
| 399 | unigene06460 | blr5828 | 5.109132983 | 4.74E-09 | flagellar basal body rod protein FlgG | up |
| 400 | unigene06461 | blr5829 | 3.569503844 | 6.98E-06 | flagellar basal body P-ring biosynthesis protein FlgA | up |
| 401 | unigene06462 | blr5830 | 5.943950887 | 3.04E-10 | flagellar basal body L-ring protein | up |
| 402 | unigene06470 | blr5838 | 3.67260169 | 2.96E-06 | flagellar basal body P-ring protein | up |
| 403 | unigene06471 | blr5839 | 3.014204154 | 0.000883102 | chemotactic signal-response protein CheL | up |
| 404 | unigene06472 | blr5840 | 4.612667862 | 9.09E-07 | hypothetical protein blr5840 | up |
| 405 | unigene06474 | bll5842 | 2.09832583 | 0.002451972 | flagellar biosynthesis regulatory protein FlaF | up |
| 406 | unigene06477 | REP4199 | 2.88612705 | 6.31E-05 | methyl-accepting chemotaxis protein | up |
| 407 | unigene06478 | bll4326 | 2.815470741 | 6.31E-05 | methyl-accepting chemotaxis protein | up |
| 408 | unigene06481 | bll4323 | 2.458842792 | 0.000399646 | hypothetical protein bll4323 | up |
| 409 | unigene06538 | bll4270 | 2.258556394 | 0.001366801 | hypothetical protein bll4270 | up |
| 410 | unigene06539 | bll4269 | 2.371715603 | 0.000999243 | hypothetical protein bll4269 | up |
| 411 | unigene06540 | bll4268 | 3.075180734 | 0.000185485 | hypothetical protein bll4268 | up |
| 412 | unigene06544 | blr4263 | 3.1868068 | 0.000106056 | hypothetical protein blr4263 | up |
| 413 | unigene06545 | blr4262 | 2.145730064 | 0.002299074 | hypothetical protein blr4262 | up |
| 414 | unigene06546 | blr4261 | 2.384972594 | 0.000732774 | hypothetical protein blr4261 | up |
| 415 | unigene06547 | blr4260 | 2.807598592 | 0.000222949 | hypothetical protein blr4260 | up |
| 416 | unigene06550 | blr4257 | 2.882945296 | 4.63E-05 | hydrolase | up |
| 417 | unigene06574 | bll0882 | 2.398667577 | 0.000811601 | hypothetical protein bll0882 | up |
| 418 | unigene06603 | bll0910 | 2.266110908 | 0.001252054 | penicillin-binding protein | up |
| 419 | unigene06760 | blr2472 | 2.568741307 | 0.000290962 | hypothetical protein blr2472 | up |
| 420 | unigene06822 | blr5602 | 2.656974198 | 0.000166428 | hippurate hydrolase | up |
| 421 | unigene06823 | blr5603 | 2.279078431 | 0.001296189 | amidase | up |
| 422 | unigene06951 | bll4076 | 2.798376595 | 7.12E-05 | 50S ribosomal protein L9 | up |
| 423 | unigene07018 | blr0382 | 2.498658421 | 0.000515 | transcriptional regulator | up |
| 424 | unigene07019 | bll0381 | 4.277769787 | 1.26E-08 | ABC transporter substrate-binding protein | up |
| 425 | unigene07020 | bll0380 | 4.211358696 | 3.62E-08 | ABC transporter ATP-binding protein | up |
| 426 | unigene07021 | bll0379 | 5.085060422 | 4.10E-10 | ABC transporter permease | up |
| 427 | unigene07022 | bll0378 | 4.30885463 | 1.05E-08 | dihydroxy-acid dehydratase | up |
| 428 | unigene07023 | bll0377 | 5.444771928 | 1.98E-11 | hypothetical protein bll0377 | up |
| 429 | unigene07106 | bll5500 | 2.136160599 | 0.002516442 | hypothetical protein bll5500 | up |
| 430 | unigene07109 | bll5503 | 2.462731001 | 0.00296923 | hypothetical protein bll5503 | up |
| 431 | unigene07171 | bll5679 | 4.528017043 | 3.96E-08 | hypothetical protein bll5679 | up |
| 432 | unigene07280 | bll8156 | 2.860496759 | 8.35E-05 | hypothetical protein bll8156 | up |
| 433 | unigene07303 | blr8135 | 2.310884743 | 0.001513731 | hypothetical protein blr8135 | up |
| 434 | unigene07308 | blr8130 | 2.373344474 | 0.002567325 | hypothetical protein blr8130 | up |
| 435 | unigene07360 | blr2041 | 2.449136074 | 0.002517285 | hypothetical protein blr2041 | up |
| 436 | unigene07362 | blr8175 | 2.387123753 | 0.001857781 | oxidoreductase | up |
| 437 | unigene07370 | bll2012 | 4.029971413 | 2.88E-06 | hypothetical protein BJ6T_78410 | up |
| 438 | unigene07404 | blr3531 | 2.232917343 | 0.003136049 | hypothetical protein blr3531 | up |
| 439 | unigene07427 | bll7571 | 3.051780366 | 0.000214333 | exoQ-like protein | up |
| 440 | unigene07499 | blr3881 | 2.485030796 | 0.000797962 | hypothetical protein blr3881 | up |
| 441 | unigene07500 | blr3880 | 2.367767142 | 0.000722039 | guanine deaminase | up |
| 442 | unigene07525 | bll2092 | 2.521291831 | 0.000492526 | para-aminobenzoate synthase component I | up |
| 443 | unigene07697 | bll1877 | 2.601659089 | 0.003147934 | hypothetical protein bll1877 | up |
| 444 | unigene07769 | —— | 5.969974472 | 0.001408973 | ABC transporter ATP-binding protein | up |
| 445 | unigene07788 | bll1804 | 2.760087605 | 0.001734035 | hypothetical protein bll1804 | up |
| 446 | unigene07789 | blr1806 | 2.240990899 | 0.001632039 | hypothetical protein blr1806 | up |
| 447 | unigene07831 | bll5848 | 2.051002806 | 0.003070728 | decarboxylase | up |
| 448 | unigene07835 | bll5852 | 4.154456356 | 6.99E-08 | hypothetical protein bll5852 | up |
| 449 | unigene07836 | bll5853 | 4.620939729 | 1.01E-08 | hypothetical protein bll5853 | up |
| 450 | unigene07837 | bll5854 | 4.917016668 | 4.37E-10 | hypothetical protein bll5854 | up |
| 451 | unigene07983 | bll1715 | 2.250309 | 0.000743679 | two component regulator | up |
| 452 | unigene07984 | bll1716 | 3.246694 | 2.60E-05 | Member of the two-component regulatory system nodV/nodW | up |
| 453 | unigene08023 | blr2476 | 2.425454495 | 0.000580544 | hypothetical protein blr2476 | up |
| 454 | unigene08025 | blr2474 | 2.564183511 | 0.000213999 | hypothetical protein blr2474 | up |
| 455 | unigene08043 | bll0393 | 3.886127 | 6.31E-05 | chemotaxis two-component hybrid sensor and regulator CheA | up |
| 456 | unigene08045 | bll0326 | 2.2862107 | 0.00192648 | methyl-accepting chemotaxis protein | up |
| 457 | unigene08055 | —— | 2.475428733 | 0.000451928 | flagellin | up |

**Table S2. The DEGs (differentially expressed genes) were identified in SREs-4222**

| NO． | Unigene | Locus ID | Log2（folds） | p-value | Description | Up/Down |
| --- | --- | --- | --- | --- | --- | --- |
|  |  |  |  |  |  | -regulation |
| 1 | unigene00114 | blr2742 | -2.36655 | 0.00120826 | hypothetical protein blr2742 | down |
| 2 | unigene00121 | bll6540 | -2.83228 | 0.0000861 | oxidoreductase | down |
| 3 | unigene00143 | blr2761 | -2.96427 | 0.0000253 | hypothetical protein blr2761 | down |
| 4 | unigene00144 | blr2762 | -2.63606 | 0.000152719 | hypothetical protein blr2762 | down |
| 5 | unigene00145 | blr2763 | -2.47196 | 0.000337033 | cbb3-type cytochrome c oxidase subunit I | down |
| 6 | unigene00146 | blr2764 | -2.94682 | 0.0000279 | cytochrome-c oxidase | down |
| 7 | unigene00147 | bsr2765 | -3.49322 | 0.00000282 | cbb3 oxidase subunit IV | down |
| 8 | unigene00148 | blr2766 | -3.40514 | 0.00000218 | cbb3 oxidase subunit III | down |
| 9 | unigene00149 | blr2767 | -2.25928 | 0.000960157 | (Fe-S)-binding protein | down |
| 10 | unigene00150 | blr2768 | -3.00889 | 0.0000218 | nitrogen fixation protein FixH | down |
| 11 | unigene00151 | blr2769 | -3.30632 | 0.00000375 | E1-E2 type cation ATPase | down |
| 12 | unigene00152 | bsr2770 | -3.68028 | 0.00000287 | FixS protein | down |
| 13 | unigene00196 | blr2806 | -5.65578 | 1.71E-10 | nitrate transporter | down |
| 14 | unigene00242 | —— | -3.04854 | 0.0000898 | hypothetical protein bll2471 | down |
| 15 | unigene00249 | blr2857 | -2.38937 | 0.001107064 | hypothetical protein BJ6T_69110 | down |
| 16 | unigene00379 | blr2987 | -3.38783 | 0.00000249 | hypothetical protein blr2987 | down |
| 17 | unigene00436 | bll3037 | -2.16479 | 0.001666026 | hypothetical protein bll3037 | down |
| 18 | unigene00470 | bll3069 | -2.24075 | 0.001080363 | SufE-like protein | down |
| 19 | unigene00547 | blr3127 | -2.20402 | 0.001287082 | heme lyase subunit CcmF | down |
| 20 | unigene00552 | Nham_1253 | -2.37696 | 0.000591823 | hypothetical protein BJ6T_48300 | down |
| 21 | unigene00581 | blr2668 | -2.72572 | 0.000150016 | hypothetical protein S23_19630 | down |
| 22 | unigene00582 | bll2662 | -2.33313 | 0.00082018 | putative arylsulfatase | down |
| 23 | unigene00642 | —— | -2.30682 | 0.000823052 | hypothetical protein | down |
| 24 | unigene00653 | blr1216 | -2.27794 | 0.000922255 | ferric uptake transcriptional regulator | down |
| 25 | unigene00882 | blr7797 | -2.12468 | 0.001891165 | RNA polymerase sigma-E factor (Sigma-24) protein | down |
| 26 | unigene00895 | —— | -3.26347 | 0.00000571 | hypothetical protein | down |
| 27 | unigene00896 | blr7780 | -2.59013 | 0.000195876 | hypothetical protein blr7780 | down |
| 28 | unigene00937 | blr7740 | -2.46431 | 0.000350599 | small heat shock protein | down |
| 29 | unigene00959 | msr6861 | -2.22715 | 0.001427635 | hypothetical protein | down |
| 30 | unigene01010 | —— | -2.8196 | 0.000135897 | hypothetical protein | down |
| 31 | unigene01032 | blr7544 | -3.03713 | 0.000017 | cytochrome c550 | down |
| 32 | unigene01071 | bll7506 | -2.31966 | 0.000886747 | putative alpha/beta hydrolase superfamily protein | down |
| 33 | unigene01185 | Rpal_4799 | -2.62251 | 0.000863403 | hypothetical protein | down |
| 34 | unigene01232 | —— | -3.53349 | 0.00000105 | hypothetical protein BJ6T_25890 | down |
| 35 | unigene01307 | BBta_6327 | -2.47739 | 0.00049854 | hypothetical protein S23_15410 | down |
| 36 | unigene01409 | bsl6675 | -3.21728 | 0.000681598 | hypothetical protein bsl6675 | down |
| 37 | unigene01411 | —— | -2.5489 | 0.000255655 | hypothetical protein bll6673 | down |
| 38 | unigene01564 | bsr6520 | -2.60278 | 0.000193459 | hypothetical protein S23_18160 | down |
| 39 | unigene01565 | bsr6521 | -2.36131 | 0.00061778 | hypothetical protein bsr6521 | down |
| 40 | unigene01592 | —— | -2.86863 | 0.001947577 | transcriptional regulator | down |
| 41 | unigene01618 | bll6468 | -2.67699 | 0.000131889 | hypothetical protein bll6468 | down |
| 42 | unigene01630 | blr6458 | -2.30404 | 0.001100806 | glucose-1-phosphate adenylyltransferease | down |
| 43 | unigene01632 | —— | -4.98678 | 1.46E-10 | hypothetical protein, partial | down |
| 44 | unigene01713 | —— | -2.84035 | 0.001306465 | hypothetical protein | down |
| 45 | unigene01735 | bll4549 | -2.40973 | 0.000524922 | isochorismatase hydrolase | down |
| 46 | unigene01810 | blr3488 | -2.72019 | 0.000577643 | hypothetical protein BJ6T_34200 | down |
| 47 | unigene01811 | blr3487 | -3.16943 | 0.0000123 | hypothetical protein BJ6T_34190 | down |
| 48 | unigene01812 | blr3486 | -3.10628 | 0.0000142 | hypothetical protein S23_35940 | down |
| 49 | unigene01815 | —— | -3.13992 | 0.0000207 | hypothetical protein | down |
| 50 | unigene01816 | blr3483 | -2.9124 | 0.0000372 | hypothetical protein BJ6T_34150 | down |
| 51 | unigene01817 | blr3482 | -3.07288 | 0.0000222 | hypothetical protein blr3482 | down |
| 52 | unigene01818 | bll3481 | -3.28872 | 0.00000433 | hypothetical protein bll3481 | down |
| 53 | unigene01819 | bll3480 | -2.42403 | 0.000479606 | hypothetical protein BJ6T_34120 | down |
| 54 | unigene01820 | blr3478 | -3.16958 | 0.0000127 | hypothetical protein BJ6T_34110 | down |
| 55 | unigene01821 | BPP1579 | -3.32459 | 0.00000462 | hypothetical protein | down |
| 56 | unigene01822 | blr3474 | -3.1106 | 0.0000119 | Orn/Lys/Arg family decarboxylase | down |
| 57 | unigene01826 | bll3470 | -2.40829 | 0.001268639 | hypothetical protein BJ6T_34060 | down |
| 58 | unigene01827 | bll3469 | -2.60478 | 0.000504974 | hypothetical protein BJ6T_34050 | down |
| 59 | unigene02121 | bll7007 | -2.31102 | 0.000785145 | oxidoreductase | down |
| 60 | unigene02132 | —— | -3.01064 | 0.000139731 | hypothetical protein | down |
| 61 | unigene02135 | bll7033 | -2.28497 | 0.001408252 | LuxA | down |
| 62 | unigene02136 | bsr7036 | -2.19109 | 0.001728085 | periplasmic nitrate reductase | down |
| 63 | unigene02137 | BBta_1863 | -2.26864 | 0.000942517 | nitrate reductase | down |
| 64 | unigene02138 | blr7038 | -2.44496 | 0.000384338 | periplasmic nitrate reductase large subunit precursor | down |
| 65 | unigene02139 | blr7039 | -2.92576 | 0.0000326 | periplasmic nitrate reductase small subunit | down |
| 66 | unigene02140 | blr7040 | -2.80747 | 0.0000627 | cytochrome C-type protein | down |
| 67 | unigene02147 | bll7046 | -2.28363 | 0.000996973 | hypothetical protein bll7046 | down |
| 68 | unigene02178 | bll7083 | -3.85811 | 0.000000141 | hypothetical protein BJ6T_23310 | down |
| 69 | unigene02180 | bsl7085 | -5.88344 | 5.07E-13 | hybrid cluster protein-associated redox disulfide domain containing protein | down |
| 70 | unigene02181 | bll7086 | -3.38862 | 0.00000225 | anaerobic coproporphyrinogen III oxidase | down |
| 71 | unigene02182 | —— | -2.94571 | 0.0000475 | hypothetical protein | down |
| 72 | unigene02183 | blr7089 | -5.8232 | 3.99E-13 | respiratory nitrite reductase | down |
| 73 | unigene02184 | blr7090 | -5.46956 | 7.53E-12 | periplasmic nitrate reductase | down |
| 74 | unigene02414 | blr7331 | -2.83134 | 0.0000644 | hypothetical protein | down |
| 75 | unigene02415 | blr7332 | -2.20385 | 0.001461537 | inosine-5'-monophosphate dehydrogenase | down |
| 76 | unigene02427 | blr7345 | -3.02557 | 0.0000189 | hypothetical protein blr7345 | down |
| 77 | unigene02428 | RPB_4120 | -2.4969 | 0.000564373 | hypothetical protein S23_59480 | down |
| 78 | unigene02642 | bll4983 | -2.55332 | 0.000224775 | hypothetical protein bll4983 | down |
| 79 | unigene02751 | bll4882 | -3.98085 | 9.19E-08 | hypothetical protein BJ6T_48160 | down |
| 80 | unigene02753 | BBta_4534 | -4.83798 | 3.14E-10 | putative TonB-dependent receptor protein | down |
| 81 | unigene02754 | BBta_4533 | -3.67495 | 0.000000507 | glycosyl hydrolase | down |
| 82 | unigene02755 | BBta_4532 | -3.96823 | 0.00000017 | hypothetical protein BJ6T_48190 | down |
| 83 | unigene02756 | bll4880 | -3.20541 | 0.00000719 | hypothetical protein S23_33030 | down |
| 84 | unigene02757 | —— | -2.53275 | 0.000309681 | hypothetical protein BJ6T_48210 | down |
| 85 | unigene02758 | bll4879 | -2.64629 | 0.000174354 | hypothetical protein BJ6T_48220 | down |
| 86 | unigene02852 | bll4785 | -2.7865 | 0.0000722 | transcriptional regulator | down |
| 87 | unigene02853 | bll4784 | -3.43584 | 0.0000017 | aldehyde dehydrogenase | down |
| 88 | unigene02854 | M446_4745 | -2.61078 | 0.000296497 | hypothetical protein BJ6T_49270 | down |
| 89 | unigene03154 | —— | -2.34752 | 0.001337513 | hypothetical protein | down |
| 90 | unigene03256 | blr1404 | -2.25927 | 0.000957618 | ATP-dependent protease, ATP-binding subunit | down |
| 91 | unigene03356 | blr1492 | -2.13682 | 0.001766177 | hypothetical protein | down |
| 92 | unigene03418 | bll7696 | -3.4037 | 0.00000267 | transcriptional regulator FixK | down |
| 93 | unigene03540 | bll5315 | -3.84379 | 0.000000155 | host cell attachment protein | down |
| 94 | unigene03758 | blr4188 | -2.97864 | 0.0000404 | cupin | down |
| 95 | unigene03938 | BCAM1782 | -2.30418 | 0.001198854 | ester cyclase | down |
| 96 | unigene04106 | blr3217 | -6.93407 | 8.01E-16 | NorD protein | down |
| 97 | unigene04107 | blr3216 | -7.07357 | 2.37E-16 | NorQ protein | down |
| 98 | unigene04108 | blr3215 | -7.27004 | 2.62E-17 | nitric oxide reductase subunit B | down |
| 99 | unigene04109 | blr3214 | -7.68736 | 2.12E-18 | nitric oxide reductase subunit C | down |
| 100 | unigene04110 | bsr3213 | -6.63378 | 0.0000964 | hypothetical protein S23_47840 | down |
| 101 | unigene04111 | BRADO1773 | -6.01669 | 1.19E-12 | hypothetical protein BJ6T_65400 | down |
| 102 | unigene04112 | blr3212 | -5.90196 | 4.27E-12 | nitric oxide reductase subunit E | down |
| 103 | unigene04143 | BBta_3130 | -2.59826 | 0.00020802 | hypothetical protein S23_48210 | down |
| 104 | unigene04200 | bll6222 | -3.76402 | 0.000000327 | Sec-independent protein translocase protein | down |
| 105 | unigene04500 | blr0497 | -3.20308 | 0.00000659 | hypothetical protein | down |
| 106 | unigene04653 | —— | -2.59667 | 0.000322665 | hypothetical protein | down |
| 107 | unigene04705 | blr1309 | -2.27511 | 0.000903044 | acetyl-coenzyme A synthetase | down |
| 108 | unigene04707 | blr1311 | -2.49659 | 0.000311054 | membrane protein | down |
| 109 | unigene04999 | bll5661 | -2.15324 | 0.001692917 | hypothetical protein | down |
| 110 | unigene05005 | bll5655 | -3.55905 | 0.000000827 | alcohol dehydrogenase | down |
| 111 | unigene05010 | bll5650 | -2.93601 | 0.0000391 | hypothetical protein BJ6T_40830 | down |
| 112 | unigene05011 | bll5649 | -2.82848 | 0.0000899 | ABC transporter ATP-binding protein | down |
| 113 | unigene05012 | bll5648 | -2.41915 | 0.00066141 | mannose-1-phosphate guanyltransferase | down |
| 114 | unigene05127 | bll3785 | -2.39443 | 0.000601308 | cytochrome C oxidase | down |
| 115 | unigene05128 | blr3787 | -2.72918 | 0.000103833 | hypothetical protein BJ6T_60770 | down |
| 116 | unigene05162 | —— | -3.27092 | 0.0000249 | hypothetical protein | down |
| 117 | unigene05163 | blr3815 | -2.77553 | 0.0000735 | cation-transporting ATPase | down |
| 118 | unigene05281 | blr2611 | -3.41197 | 0.00000208 | hypothetical protein blr2611 | down |
| 119 | unigene05286 | blr2607 | -2.31073 | 0.000803389 | hypothetical protein BJ6T_71970 | down |
| 120 | unigene05303 | —— | -3.23577 | 0.00000806 | hypothetical protein | down |
| 121 | unigene05304 | —— | -3.14385 | 0.0000203 | hypothetical protein | down |
| 122 | unigene05305 | —— | -2.16283 | 0.001570224 | hypothetical protein | down |
| 123 | unigene05319 | bll2590 | -3.61454 | 0.000000631 | hypothetical protein bll2590 | down |
| 124 | unigene05320 | —— | -2.74261 | 0.000271371 | hypothetical protein S23_53920 | down |
| 125 | unigene05397 | bll2518 | -3.27816 | 0.00000471 | phosphoketolase | down |
| 126 | unigene05398 | bll2517 | -3.54561 | 0.00000253 | putative acetate kinase (acetokinase) protein | down |
| 127 | unigene05467 | bll0759 | -2.8888 | 0.0000404 | phosphopantothenate synthase | down |
| 128 | unigene05546 | bll0687 | -2.29069 | 0.00085913 | transcriptional regulatory protein AraC family | down |
| 129 | unigene05607 | blr6074 | -2.76049 | 0.0000753 | hypothetical protein blr6074 | down |
| 130 | unigene05609 | bll6073 | -3.12356 | 0.0000104 | poly-beta-hydroxybutyrate polymerase | down |
| 131 | unigene05611 | blr6071 | -2.59847 | 0.000238114 | DNA-binding protein | down |
| 132 | unigene05612 | blr6070 | -2.42012 | 0.000589792 | alcohol dehydrogenase | down |
| 133 | unigene05613 | bll6069 | -3.09463 | 0.0000122 | universal stress protein UspA | down |
| 134 | unigene05614 | bll6068 | -2.88556 | 0.00004 | hypothetical protein bll6068 | down |
| 135 | unigene05615 | BBta_5752 | -2.89312 | 0.0000447 | hypothetical protein blr6067 | down |
| 136 | unigene05616 | —— | -3.24062 | 0.00000585 | hypothetical protein bsr6066 | down |
| 137 | unigene05617 | bll6065 | -2.40017 | 0.000506739 | ABC transporter permease | down |
| 138 | unigene05618 | bll6064 | -2.85944 | 0.0000507 | iron ABC transporter ATP-binding protein | down |
| 139 | unigene05619 | bll6063 | -3.29506 | 0.00000436 | hypothetical protein | down |
| 140 | unigene05748 | Nham_1177 | -2.901 | 0.000039 | co-chaperonin GroES | down |
| 141 | unigene05805 | bll0225 | -2.51342 | 0.000273848 | acetoacetyl CoA reductase | down |
| 142 | unigene05821 | bsr0210 | -2.26659 | 0.000989686 | glutaredoxin | down |
| 143 | unigene05917 | bll6909 | -2.18328 | 0.001618274 | hypothetical protein bll6909 | down |
| 144 | unigene05944 | bsl5479 | -2.9582 | 0.00138806 | hypothetical protein bsl5479 | down |
| 145 | unigene05945 | bll5480 | -2.29039 | 0.001230224 | chaperone | down |
| 146 | unigene05954 | —— | -3.11171 | 0.0000178 | _ | down |
| 147 | unigene06057 | bsr3925 | -2.2127 | 0.001334316 | hypothetical protein | down |
| 148 | unigene06186 | bll2310 | -2.21203 | 0.001245928 | putative formate dehydrogenase accessory protein | down |
| 149 | unigene06472 | blr4759 | -2.18383 | 0.001418642 | heme biosynthesis protein HemY | down |
| 150 | unigene06510 | bsr4721 | -2.34788 | 0.000728862 | hypothetical protein bsr4721 | down |
| 151 | unigene06512 | bll4718 | -2.79031 | 0.000068 | hypothetical protein | down |
| 152 | unigene06548 | BRADO3989 | -2.63678 | 0.000186811 | hypothetical protein | down |
| 153 | unigene06654 | —— | -4.55705 | 2.38E-08 | hypothetical protein | down |
| 154 | unigene06655 | Xaut_0180 | -4.56188 | 1.9E-09 | hypothetical protein BJ6T_51590 | down |
| 155 | unigene06656 | —— | -2.97815 | 0.0000585 | hypothetical protein BJ6T_51580 | down |
| 156 | unigene06657 | —— | -2.81965 | 0.000671796 | hypothetical protein BJ6T_51570 | down |
| 157 | unigene06658 | bll3462 | -3.80721 | 0.000000444 | 3-oxoadipate CoA-transferase subunit A | down |
| 158 | unigene06659 | Nham_0867 | -3.79766 | 0.000000472 | 3-oxoadipate CoA-transferase subunit B | down |
| 159 | unigene06660 | bll3460 | -3.48865 | 0.00000894 | nodulation protein N | down |
| 160 | unigene06661 | blr3459 | -4.49464 | 1.19E-08 | enoyl-ACP reductase | down |
| 161 | unigene06662 | blr3458 | -3.85333 | 0.000000338 | acetate kinase | down |
| 162 | unigene06663 | blr3457 | -3.81984 | 0.000000323 | phosphate acetyltransferase | down |
| 163 | unigene06664 | blr3456 | -4.10831 | 3.35E-08 | hypothetical protein BJ6T_51500 | down |
| 164 | unigene06665 | Nham_0861 | -3.18223 | 0.000175558 | N-acetyltransferase GCN5 | down |
| 165 | unigene06666 | Nham_0883 | -4.67032 | 0.000000392 | hypothetical protein | down |
| 166 | unigene06925 | bll0330 | -2.19238 | 0.001393348 | two-component response regulator | down |
| 167 | unigene06967 | —— | -2.20063 | 0.001614424 | hypothetical protein | down |
| 168 | unigene07046 | —— | -2.53302 | 0.000430902 | hypothetical protein BJ6T_76290 | down |
| 169 | unigene07078 | blr1883 | -3.09242 | 0.0000136 | RNA polymerase factor sigma-54 | down |
| 170 | unigene07348 | —— | -2.51673 | 0.000463326 | hypothetical protein S23_69380 | down |
| 171 | unigene07403 | —— | -3.26133 | 0.00000916 | hypothetical protein | down |
| 172 | unigene07413 | —— | -3.09824 | 0.0000254 | _ | down |
| 173 | unigene07414 | blr6128 | -2.6488 | 0.000151468 | cytochrome C552 | down |
| 174 | unigene07445 | blr5540 | -2.37039 | 0.000564543 | NAD(P)H nitroreductase | down |
| 175 | unigene07505 | bll2007 | -3.00699 | 0.0000221 | coproporphyrinogen III oxidase | down |
| 176 | unigene07550 | bll7982 | -2.8637 | 0.0000435 | hypothetical protein bll7982 | down |
| 177 | unigene07551 | bll7981 | -2.95363 | 0.0000269 | dehydrogenase | down |
| 178 | unigene07564 | RPD_3899 | -2.65333 | 0.000186614 | hypothetical protein | down |
| 179 | unigene07581 | —— | -2.4193 | 0.000950893 | hypothetical protein | down |
| 180 | unigene07649 | bll8143 | -2.58971 | 0.000219419 | pyridoxamine 5'-phosphate oxidase | down |
| 181 | unigene07772 | bll2665 | -2.91907 | 0.0000527 | arylsulfatase | down |
| 182 | unigene07784 | bll1766 | -3.25088 | 0.0000111 | outer membrane protein | down |
| 183 | unigene07929 | —— | -2.43179 | 0.000526915 | hypothetical protein BJ6T_76910 | down |
| 184 | unigene07955 | —— | -3.67838 | 0.000000665 | ID838 | down |
| 185 | unigene08143 | —— | -2.9424 | 0.0000588 | transcriptional regulator | down |
| 186 | unigene00207 | blr2815 | 2.930227 | 0.000037 | transketolase | up |
| 187 | unigene00321 | BBta_2886 | 2.592156 | 0.000573277 | ABC transporter permease | up |
| 188 | unigene00322 | blr2925 | 3.051806 | 0.0000792 | ABC transporter | up |
| 189 | unigene00323 | blr2926 | 2.607402 | 0.000420291 | amino acid ABC transporter ATP-binding protein | up |
| 190 | unigene00330 | blr2934 | 2.281161 | 0.001358195 | cation efflux protein | up |
| 191 | unigene00815 | —— | 3.188448 | 0.000212047 | hypothetical protein S23_09270 | up |
| 192 | unigene01119 | bll7458 | 2.587323 | 0.000310137 | hypothetical protein BJ6T_18320 | up |
| 193 | unigene01158 | —— | 3.306381 | 0.00000888 | hypothetical protein | up |
| 194 | unigene01159 | —— | 3.557295 | 0.00000105 | hypothetical protein S23_60470 | up |
| 195 | unigene01187 | bll6864 | 4.77693 | 5.81E-09 | flagellar M-ring protein FliF | up |
| 196 | unigene01188 | bll6863 | 5.609649 | 7.63E-09 | hypothetical protein | up |
| 197 | unigene01189 | bll6862 | 5.457411 | 1.77E-09 | flagellar motor protein MotB | up |
| 198 | unigene01190 | bll6861 | 4.221919 | 0.00000641 | motC | up |
| 199 | unigene01191 | bll6860 | 4.293769 | 0.000000248 | hypothetical protein bll6860 | up |
| 200 | unigene01192 | bll6859 | 4.873541 | 0.0000225 | hypothetical protein S23_14140 | up |
| 201 | unigene01193 | bll6858 | 4.81032 | 8.59E-10 | flagellar hook protein FlgE | up |
| 202 | unigene01194 | bll6857 | 4.474037 | 1.75E-08 | flagellar hook-associated protein FlgK | up |
| 203 | unigene01195 | bll6856 | 4.627045 | 8.8E-09 | flagellar hook-associated protein FlgL | up |
| 204 | unigene01196 | bll6855 | 7.839782 | 0.000000194 | flagellar assembly protein | up |
| 205 | unigene01197 | bll6854 | 4.357872 | 0.0000171 | flagellar biosynthesis repressor FlbT | up |
| 206 | unigene01198 | bll6853 | 3.852567 | 0.00000237 | flagellar basal body rod modification protein | up |
| 207 | unigene01199 | bsl6852 | 4.625264 | 0.0000691 | flagellar biosynthesis protein FliQ | up |
| 208 | unigene01200 | bll6851 | 4.726066 | 1.24E-08 | flagellar biosynthesis protein FlhA | up |
| 209 | unigene01201 | bll6850 | 3.457767 | 0.000294417 | flagellar biosynthesis protein FliR | up |
| 210 | unigene01202 | —— | 3.630502 | 0.000395255 | hypothetical protein | up |
| 211 | unigene01203 | bll6849 | 3.724371 | 0.000336207 | hypothetical protein bll6849 | up |
| 212 | unigene01204 | bll6848 | 3.758861 | 0.0000324 | hypothetical protein bll6848 | up |
| 213 | unigene01205 | bll6847 | 3.330601 | 0.000154729 | hypothetical protein bll6847 | up |
| 214 | unigene01207 | bll6844 | 3.088305 | 0.0000541 | hypothetical protein BJ6T_25560 | up |
| 215 | unigene01875 | blr4450 | 2.660801 | 0.000200466 | hypothetical protein blr4450 | up |
| 216 | unigene01973 | blr4374 | 3.484329 | 0.0000105 | 4-hydroxythreonine-4-phosphate dehydrogenase | up |
| 217 | unigene01974 | blr4373 | 2.550358 | 0.000638926 | tricarboxylate transporter | up |
| 218 | unigene01976 | blr4371 | 2.718988 | 0.000141901 | hypothetical protein blr4371 | up |
| 219 | unigene01977 | blr4370 | 2.457195 | 0.000542598 | hypothetical protein blr4370 | up |
| 220 | unigene01978 | blr4369 | 2.200517 | 0.001929821 | enoyl-CoA hydratase | up |
| 221 | unigene02109 | blr6996 | 3.443098 | 0.00000592 | hypothetical protein blr6996 | up |
| 222 | unigene02110 | blr6997 | 2.89747 | 0.0000756 | flagellar hook assembly protein | up |
| 223 | unigene02118 | blr7005 | 2.296219 | 0.001904213 | hypothetical protein S23_13000 | up |
| 224 | unigene03061 | blr1052 | 2.323149 | 0.00076278 | ABC transporter binding protein | up |
| 225 | unigene03087 | bll1076 | 2.619914 | 0.000183582 | transcriptional regulator | up |
| 226 | unigene03088 | blr1077 | 3.326796 | 0.00000465 | acetyl-CoA synthetase | up |
| 227 | unigene03089 | blr1078 | 3.965556 | 0.000000198 | hydrolase | up |
| 228 | unigene03091 | blr1080 | 4.89219 | 2.45E-10 | hypothetical protein BJ6T_11100 | up |
| 229 | unigene03092 | blr1081 | 6.380703 | 1.23E-14 | benzoyl-CoA oxygenase component | up |
| 230 | unigene04019 | H16_A1698 | 3.312956 | 0.00000693 | hypothetical protein BJ6T_61920 | up |
| 231 | unigene04020 | blr3537 | 3.075772 | 0.0000242 | carnitinyl-CoA dehydratase | up |
| 232 | unigene04021 | blr3536 | 2.850102 | 0.000222988 | 4-chlorobenzoate--CoA ligase | up |
| 233 | unigene04024 | blr3533 | 2.216415 | 0.001855974 | carbon monoxide dehydrogenase | up |
| 234 | unigene04032 | bll3528 | 3.214525 | 0.0000144 | hypothetical protein | up |
| 235 | unigene04136 | blr3179 | 2.40175 | 0.000575172 | alanine dehydrogenase | up |
| 236 | unigene04247 | —— | 4.072962 | 0.0000032 | acyl-CoA transferase | up |
| 237 | unigene04248 | Daci_1165 | 3.877171 | 0.0000027 | isovaleryl-CoA dehydrogenase | up |
| 238 | unigene04249 | blr3454 | 4.670255 | 1.85E-08 | hypothetical protein blr3454 | up |
| 239 | unigene04250 | blr3453 | 4.332006 | 0.0000198 | hypothetical protein blr3453 | up |
| 240 | unigene04251 | blr3452 | 4.345073 | 0.000000262 | hypothetical protein blr3452 | up |
| 241 | unigene04252 | blr3451 | 3.821291 | 0.00000101 | oxidoreductase | up |
| 242 | unigene04254 | blr3450 | 3.911264 | 0.0000023 | hypothetical protein blr3450 | up |
| 243 | unigene04255 | blr3449 | 4.47883 | 0.000000332 | dehydrogenase | up |
| 244 | unigene04256 | blr3448 | 4.481935 | 3.63E-08 | acyl-CoA dehydrogenase | up |
| 245 | unigene04257 | blr3447 | 4.821369 | 1.59E-09 | substrate-binding protein | up |
| 246 | unigene04258 | blr3446 | 4.788546 | 4.39E-09 | 6-carboxyhexanoate--CoA ligase | up |
| 247 | unigene04259 | blr3445 | 4.900347 | 5.66E-10 | enoyl-CoA hydratase | up |
| 248 | unigene04262 | bll3444 | 3.847712 | 0.00000113 | hypothetical protein bll3444 | up |
| 249 | unigene04264 | bll3442 | 5.0279 | 0.0000114 | senescence marker protein-30 (SMP-30) (regucalcin) (RC) | up |
| 250 | unigene04266 | bll3440 | 4.527011 | 0.00000749 | hypothetical protein bll3440 | up |
| 251 | unigene04701 | bll1305 | 2.773903 | 0.000122214 | hypothetical protein | up |
| 252 | unigene04704 | blr1308 | 2.385308 | 0.000574753 | hypothetical protein | up |
| 253 | unigene04818 | blr5840 | 2.669485 | 0.001042881 | hypothetical protein | up |
| 254 | unigene04820 | blr5838 | 2.685993 | 0.000311672 | flagellar basal body P-ring protein | up |
| 255 | unigene04830 | blr5830 | 2.545 | 0.000742713 | flagellar basal body L-ring protein | up |
| 256 | unigene04831 | blr5829 | 2.272604 | 0.001903546 | flagellar basal body P-ring biosynthesis protein FlgA | up |
| 257 | unigene04832 | blr5828 | 2.603936 | 0.000442878 | flagellar basal body rod protein FlgG | up |
| 258 | unigene04833 | blr5827 | 2.988514 | 0.0000769 | flagellar basal body rod protein FlgF | up |
| 259 | unigene04979 | bll5679 | 2.531095 | 0.000538607 | hypothetical protein | up |
| 260 | unigene05873 | bll6867 | 4.678384 | 0.00000349 | export apparatus protein | up |
| 261 | unigene05874 | bll6868 | 7.47901 | 0.00000145 | flagellar protein | up |
| 262 | unigene05875 | bll6869 | 3.906764 | 0.00000168 | flagellar L-ring protein FlgH | up |
| 263 | unigene05876 | bll6870 | 5.465572 | 3.97E-08 | hypothetical protein BJ6T_25280 | up |
| 264 | unigene05877 | bll6871 | 4.288007 | 0.000000363 | flagellar basal-body P-ring protein | up |
| 265 | unigene05878 | bll6872 | 4.57531 | 0.000000929 | flagellar basal body P-ring biosynthesis protein FlgA | up |
| 266 | unigene05879 | bll6873 | 4.726877 | 3.57E-09 | flagellar basal body rod protein FlgG | up |
| 267 | unigene05880 | bll6874 | 4.697274 | 8.74E-08 | flagellar hook-basal body complex protein | up |
| 268 | unigene05881 | bll6875 | 9.42054 | 1.39E-11 | flagellar basal body rod protein FlgC | up |
| 269 | unigene05882 | bll6876 | 4.399633 | 0.000000134 | flagellar basal-body rod protein FlgB | up |
| 270 | unigene05883 | bll6877 | 4.241303 | 0.00000128 | flagellar biosynthetic protein | up |
| 271 | unigene05884 | bll6878 | 4.394419 | 9.48E-08 | flagellar motor switch protein FliG | up |
| 272 | unigene05885 | bll6879 | 3.019135 | 0.000467857 | flagellar motor switch protein FliN | up |
| 273 | unigene05886 | —— | 3.752868 | 0.000078 | hypothetical protein | up |
| 274 | unigene05887 | bll6881 | 3.575473 | 0.00000722 | flagellar motor switch protein FliM | up |
| 275 | unigene05888 | bll6882 | 3.664678 | 0.00000373 | chemotaxis protein MotA | up |
| 276 | unigene05889 | blr6883 | 4.549851 | 2.94E-08 | hypothetical protein blr6883 | up |
| 277 | unigene05890 | blr6884 | 4.386438 | 0.000000209 | flagellar basal body rod protein FlgF | up |
| 278 | unigene05891 | blr6885 | 3.682069 | 0.0000116 | flagellum-specific ATP synthase | up |
| 279 | unigene05920 | blr2689 | 4.170887 | 0.000000369 | benzoylformate decarboxylase | up |
| 280 | unigene05921 | blr2688 | 6.750471 | 1.18E-12 | vanillin: NAD oxidoreductase | up |
| 281 | unigene05922 | blr2687 | 5.213878 | 7.89E-09 | metabolite transport protein | up |
| 282 | unigene05923 | blr2686 | 5.100727 | 2.58E-09 | hypothetical protein blr2686 | up |
| 283 | unigene06678 | blr5623 | 2.306988 | 0.001084143 | hypothetical protein blr5623 | up |
| 284 | unigene06789 | bll5852 | 3.164014 | 0.0000167 | hypothetical protein bll5852 | up |
| 285 | unigene06790 | bll5853 | 3.529698 | 0.00000405 | hypothetical protein BJ6T_38540 | up |
| 286 | unigene06791 | bll5854 | 3.210922 | 0.00000769 | hypothetical protein BJ6T_38530 | up |
| 287 | unigene06977 | BBta_6492 | 3.233639 | 0.0000106 | hypothetical protein BJ6T_03340 | up |
| 288 | unigene06978 | bll0377 | 2.196142 | 0.001701756 | hypothetical protein BJ6T_03350 | up |
| 289 | unigene07199 | blr2386 | 2.377717 | 0.001309772 | two-component response regulator | up |
| 290 | unigene07259 | blr2442 | 4.00039 | 5.73E-08 | ABC transporter substrate-binding protein | up |
| 291 | unigene07410 | bll6131 | 2.67393 | 0.000997073 | hypothetical protein bll6131 | up |
| 292 | unigene07880 | blr5947 | 2.335716 | 0.00098894 | hypothetical protein | up |
| 293 | unigene08173 | blr1676 | 2.263218 | 0.001148209 | hypothetical protein blr1676 | up |

**Table S3. The genes were related to** **the “two-component system”, which was found to be significantly enriched in the KEGG analysis in *B. diazoefficiens* 4534**

| NO． | Unigene | Locus ID | Log2（folds） | p-value | Description | Up/Down  -regulation |
| --- | --- | --- | --- | --- | --- | --- |
| 1 | unigene 00047 | blr2548 | 2.111788 | 0.002262826 | chemotaxis protein | up |
| 2 | unigene 00442 | blr2931 | 3.006625 | 2.43E-05 | methyl-accepting chemotaxis protein | up |
| 3 | unigene 00982 | bll3462 | -2.48414 | 0.000869768 | 3-oxoadipate CoA-transferase subunit A | down |
| 4 | unigene 01735 | bll6866 | 2.429321 | 0.000901749 | flagellin | up |
| 5 | unigene 01736 | bll6865 | 2.342994 | 0.000947994 | flagellin | up |
| 6 | unigene03912 | blr7797 | 2.604028 | 0.000250318 | RNA polymerase sigma factor σ^EcfG^ | up |
| 7 | unigene 04673 | blr2194 | 2.432272 | 0.000654037 | chemotaxis two-component response regulator | up |
| 8 | unigene 04674 | blr2195 | 2.434486 | 0.000552986 | chemotaxis-specific methylesterase | up |
| 9 | unigene03913 | bll7795 | 3.743454 | 0.00013463 | two-component response regulator *phyR* | up |
| 10 | unigene 04733 | blr2247 | 2.530566 | 0.001335432 | fatty acid desaturase | up |
| 11 | unigene 04830 | blr2345 | 2.803495 | 7.24E-05 | methyl-accepting chemotaxis protein | up |
| 12 | unigene 04828 | blr2343 | 2.328492 | 0.000778398 | chemotaxis protein | up |
| 13 | unigene07984 | bll1714 | 3.246694 | 2.60E-05 | Member of the two-component regulatory system nodV/nodW | up |
| 14 | unigene 04831 | blr2346 | 2.826547 | 0.000268124 | chemotaxis protein | up |
| 15 | unigene 04833 | blr2348 | 4.255502 | 1.30E-07 | protein-glutamate O-methyltransferase | up |
| 16 | unigene 05037 | bll7954 | 2.035915 | 0.003032327 | methyl accepting chemotaxis protein | up |
| 17 | unigene 06478 | bll4326 | 2.815471 | 6.31E-05 | methyl-accepting chemotaxis protein | up |
| 18 | unigene 07983 | bll1715 | 2.250309 | 0.000743679 | two component regulator | up |
| 19 | unigene 07984 | bll1716 | 2.972592 | 0.001035801 | two component regulator | up |

**Table S4. The genes were related to the “bacterial chemotaxis”, which was found to be significantly enriched in the KEGG analysis in *B. diazoefficiens* 4534**

| NO． | Unigene | Locus ID | Log2（folds） | p-value | Description | Up/Down  -regulation |
| --- | --- | --- | --- | --- | --- | --- |
| 1 | unigene01739 | bll6862 | 2.780351 | 0.001043 | flagellar motor protein MotB | up |
| 2 | unigene00442 | blr2931 | 3.006625 | 2.43E-05 | methyl-accepting chemotaxis protein | up |
| 3 | unigene04832 | blr2347 | 2.826547 | 0.000268124 | chemotaxis protein | up |
| 4 | unigene01723 | bll6878 | 2.572905 | 0.001516278 | flagellar motor switch protein | up |
| 5 | unigene01720 | bll6881 | 3.322858 | 0.000469533 | hypothetical protein bll6881 | up |
| 6 | unigene08043 | bll0393 | 3.886127 | 6.31E-05 | chemotaxis two-component hybrid sensor and regulator CheA | up |
| 7 | unigene05037 | bll7954 | 2.035915 | 0.003032327 | methyl accepting chemotaxis protein | up |
| 8 | unigene04673 | blr2194 | 2.432272 | 0.000654037 | chemotaxis two-component response regulator | up |
| 9 | unigene00047 | blr2548 | 2.111788 | 0.002262826 | chemotaxis protein | up |
| 10 | unigene04830 | blr2345 | 2.803495 | 7.24E-05 | methyl-accepting chemotaxis protein | up |
| 11 | unigene04831 | blr2346 | 2.826547 | 0.000268124 | chemotaxis protein | up |
| 12 | unigene04674 | blr2195 | 2.434486 | 0.000552986 | chemotaxis-specific methylesterase | up |
| 13 | unigene05459 | blr3801 | 2.220126 | 0.001626843 | chemotaxis protein | up |
| 14 | unigene04833 | blr2348 | 4.255502 | 1.30E-07 | protein-glutamate O-methyltransferase | up |
| 15 | unigene00183 | bll2674 | 2.776179 | 9.39E-05 | sugar ABC transporter substrate-binding protein | up |
| 16 | unigene06477 | RPE4199 | 2.886127 | 6.31E-05 | methyl-accepting chemotaxis protein | up |
| 17 | unigene04829 | blr2344 | 3.246694 | 2.60E-05 | chemotaxis protein | up |

Table S5. The genes were related to the “ABC transporters”, which was found to be significantly enriched in the KEGG analysis in *B. diazoefficiens* 4534

| NO． | Unigene | Locus ID | Log2（folds） | p-value | Description | Up/Down  -regulation |
| --- | --- | --- | --- | --- | --- | --- |
| 1 | unigene00434 | blr2923 | 5.33981 | 2.73E-10 | amino acid ABC transporter permease | up |
| 2 | unigene00435 | blr2924 | 5.618775 | 3.16E-11 | amino acid ABC transporter permease | up |
| 3 | unigene00436 | blr2925 | 6.28541 | 1.28E-12 | amino acid ABC transporter ATP-binding protein | up |
| 4 | unigene00437 | blr2926 | 5.069266 | 5.85E-10 | amino acid ABC transporter ATP-binding protein | up |
| 5 | unigene02629 | blr1067 | 2.946649 | 0.000145 | ABC transporter ATP-binding protein | up |
| 6 | unigene00433 | blr2922 | 4.245837 | 1.49E-08 | amino acid ABC transporter substrate-binding protein | up |
| 7 | unigene02627 | blr1065 | 2.627955 | 0.000334 | ABC transporter permease | up |
| 8 | unigene02046 | bll6236 | 4.361804 | 3.68E-06 | ABC transporter substrate-binding protein | up |
| 9 | unigene02045 | bll6235 | 6.95842 | 1.80E-05 | ABC transporter permease | up |
| 10 | unigene02628 | blr1066 | 2.832054 | 0.000416 | ABC transporter ATP-binding protein | up |
| 11 | unigene02212 | bll6407 | 2.827498 | 0.000873 | ABC transporter substrate-binding protein | up |
| 12 | unigene01797 | blr6804 | 2.788788 | 9.21E-05 | substrate-binding protein | up |
| 13 | unigene00730 | blr3210 | 3.773205 | 4.09E-07 | sugar ABC transporter permease | up |
| 14 | unigene02208 | bll6403 | 5.89325 | 0.001409 | ABC transporter permease | up |
| 15 | unigene00728 | unigene00728 | 4.53455 | 2.12E-09 | sugar ABC transporter substrate-binding protein | up |
| 16 | unigene00729 | blr3209 | 3.83993 | 2.02E-07 | sugar ABC transporter ATP-binding protein | up |
| 17 | unigene02613 | blr1052 | 2.489484 | 0.000351 | ABC transporter binding protein | up |
| 18 | unigene00183 | bll2674 | 2.776179 | 9.39E-05 | sugar ABC transporter substrate-binding protein | up |
| 19 | unigene00967 | blr3447 | 2.815885 | 9.59E-05 | substrate-binding protein | up |
| 20 | unigene04758 | blr2271 | 3.169973 | 0.00017 | sugar ABC transporter permease | up |
